# Aggregation-induced emission luminogens reveal cell cycle-dependent telomerase activity in cancer cells

**DOI:** 10.1093/nsr/nwaa306

**Published:** 2021-01-02

**Authors:** Xia Wu, Jun Wu, Jun Dai, Biao Chen, Zhe Chen, Shixuan Wang, Feng Wu, Xiaoding Lou, Fan Xia

**Affiliations:** Engineering Research Center of Nano-Geomaterials of Ministry of Education, Faculty of Materials Science and Chemistry, China University of Geosciences, Wuhan 430074, China; Engineering Research Center of Nano-Geomaterials of Ministry of Education, Faculty of Materials Science and Chemistry, China University of Geosciences, Wuhan 430074, China; Department of Obstetrics and Gynecology, Tongji Hospital, Tongji Medical College, Huazhong University of Science and Technology, Wuhan 430074, China; Department of Obstetrics and Gynecology, Tongji Hospital, Tongji Medical College, Huazhong University of Science and Technology, Wuhan 430074, China; Department of Obstetrics and Gynecology, Tongji Hospital, Tongji Medical College, Huazhong University of Science and Technology, Wuhan 430074, China; Department of Obstetrics and Gynecology, Tongji Hospital, Tongji Medical College, Huazhong University of Science and Technology, Wuhan 430074, China; Engineering Research Center of Nano-Geomaterials of Ministry of Education, Faculty of Materials Science and Chemistry, China University of Geosciences, Wuhan 430074, China; Engineering Research Center of Nano-Geomaterials of Ministry of Education, Faculty of Materials Science and Chemistry, China University of Geosciences, Wuhan 430074, China; Engineering Research Center of Nano-Geomaterials of Ministry of Education, Faculty of Materials Science and Chemistry, China University of Geosciences, Wuhan 430074, China

**Keywords:** cell cycle, TERT mRNA, telomerase activity, AIEgens, biomarkers

## Abstract

Telomerase acts as an important biomarker for tumor identification, and synthesizes telomeric repeats at the end of chromosome telomeres during the replicative phase of the cell cycle; thus, the expression level of telomerase changes as the cell cycle progresses. TERT mRNA expression and telomerase activity were significantly increased in over 80% of human cancers from tissue specimens. Although many efforts have been made in detecting the activity of TERT mRNA and active telomerase, the heterogeneous behavior of the cell cycle was overlooked, which might affect the accuracy of the detection results. Herein, the AIEgen-based biosensing systems of PyTPA-DNA and Silole-R were developed to detect the cellular level of TERT mRNA and telomerase in different cell cycles. As a result, the fluorescence signal of cancer cells gradually increased from G0/G1, G1/S to S phase. In contrast, both cancer cells arrested at G2/M phase and normal cells exhibited negligible fluorescence intensities. Compared to normal tissues, malignant tumor samples demonstrated a significant turn-on fluorescence signal. Furthermore, the transcriptomics profiling revealed that tumor biomarkers changed as the cell cycle progressed and biomarkers of CA9, TK1 and EGFR were more abundantly expressed at early S stage. In this vein, our study presented advanced biosensing tools for more accurate analysis of the cell-cycle-dependent activity of TERT mRNA and active telomerase in clinical tissue samples.

## Teaser

AIEgen-based biosensing system was developed to detect telomerase in different cell cycles and the fluorescence signal of cancer cells increased from G0/G1 to S phase. While, cancer cells arrested at G2/M phase and normal cells exhibited the negligible signal.

## INTRODUCTION

Cancer is a significant cause of death worldwide and numerous efforts have been devoted to the development of methods for early detection and quantitative measurement of cancer biomarkers to obtain information correlated to tumorigenesis [1]. Activation of telomerase is a typical feature of more than 80% of immortalized cells but not detectable in normal somatic tissues [2,3]. Once telomerase is activated, a majority of tumor cells could maintain the telomere during the S phase of the cell cycle [4–6]. Telomerase is a ribonucleoprotein complex consisting of an RNA template moiety, a reverse transcriptase catalytic component (TERT protein) together with associated proteins [7]. Activation of telomerase is the principal manifestation of regulating the expression of TERT messenger RNA (TERT mRNA) [8,9]. In particular, telomerase activity can be inhibited by down-regulating TERT mRNA expression in different types of human malignancies [10,11]. Thus, TERT mRNA as well as telomerase activity were used as useful diagnostic biomarkers during tumorigenesis [12].

Many works have been reported in developing new bioanalytical methods for detection of TERT mRNA and telomerase activity [13,14]. Current detecting techniques (the golden standard) for TERT mRNA are mainly based on Real-time Quantitative Polymerase Chain Reaction (RT-qPCR) [15,16]. The standard TRAP assay (telomeric repeat amplification protocol) [3,17,18], optical detection [19,20], colorimetric assays [21], biosensor chip [22] and electrochemical strategies [23] have been developed for telomerase activity detection. Fluorescence methods have the advantage of simplicity, sensitivity and rapidity [24–26], thus they were widely used in cell-lysate-dependent detecting assays [27,28] and intracellular imaging [29–31]. Moreover, our group's prior work designed a series of fluorescent probes to light up telomerase on the basis of aggregation-induced emission luminogens (AIEgens) [32–34]. AIEgens possess many advantages such as long-term tracking ability, low background and strong resistance to photo-bleaching. They exhibit a higher fluorescence in aggregated states and have been widely applied in the field of biochemical analysis and imaging [35–38].

Although lots of methods have been developed to detect telomerase, those methods mainly relied upon the analysis of asynchronous cells with different phases of the cell cycle. The cell cycle is an integrated network that contributes towards balancing the process of various bio-macromolecule syntheses, assemblies and interactions [39–41]. Some research indicates that the telomerase activity of human tumor cells changes as the cell progresses through different stages of the cell cycle [42–44]. This means that the accurate analysis of telomerase may be affected by the different phases of the cell cycle.

**Scheme 1.**
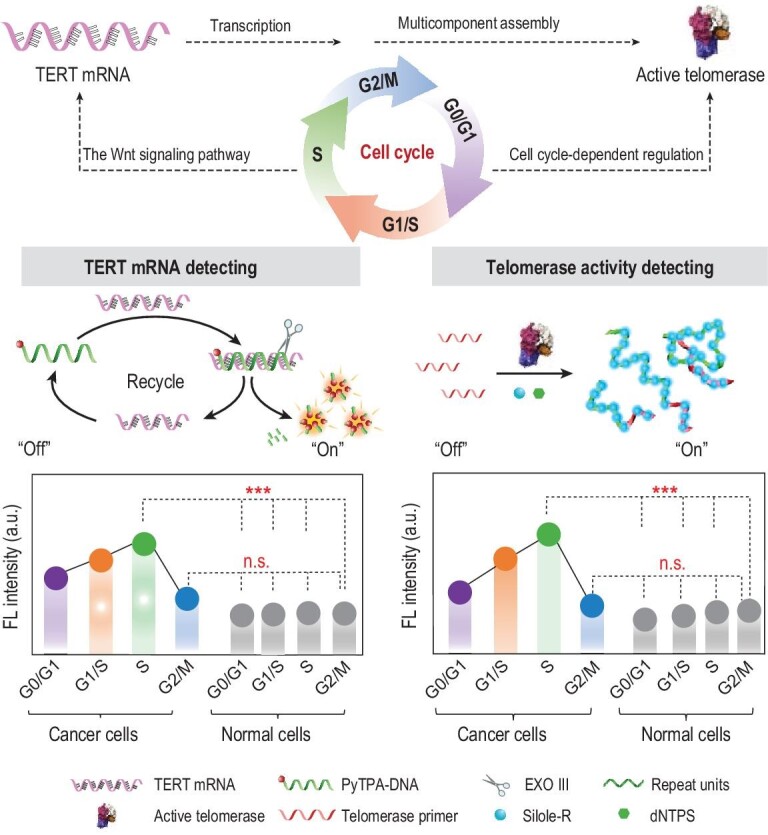
AIEgen-based fluorescence detecting system for analysis of TERT mRNA and telomerase activity in different phases of the cell cycle (G0/G1, G1/S, S and G2/M phase).

Herein, we investigated the role of cell cycle progression (G0/G1, G1/S, S and G2/M phase) in analyzing telomerase (TERT mRNA and telomerase activity) in cancer cells based on an AIEgen-based fluorescence detecting system. As shown in Scheme 1, in the absence of TERT mRNA and exonuclease III (Exo III), the redundant PyTPA-DNA probe could complement hybridization and then be cleaved by Exo III to release the target and fluorogen (PyTPA-N_3_). Moreover, in the presence of active telomerase, the template strand primer (TP) could be extended to form long negatively charged DNA chains. The positively charged AIE dye (Silole-R) was spontaneously combined to the chains via electrostatic interaction. On the basis of this system, we tested TERT mRNA and telomerase activity between different phases of cancer cells vs. normal cells. Results show that the fluorescence signal of cancer cells arrested at G0/G1, G1/S, S phase increased as the cell cycle progressed, while the cancer cells arrested at G2/M phase and normal cells showed a negligible intensity. Moreover, this fluorescence analyzing system was successfully used in the tissue samples of malignant tumors. Finally, we compared the expression levels of some biomarkers during difference phases of the cell cycle on the basis of transcriptomics profiling. These results, therefore, suggest that future studies on tumor biomarkers analysis, such as TERT mRNA and telomerase activity, should consider the phase of the cell cycle.

## RESULTS AND DISCUSSION

### Bioprobes for TERT mRNA and telomerase activity

**Figure 1.**
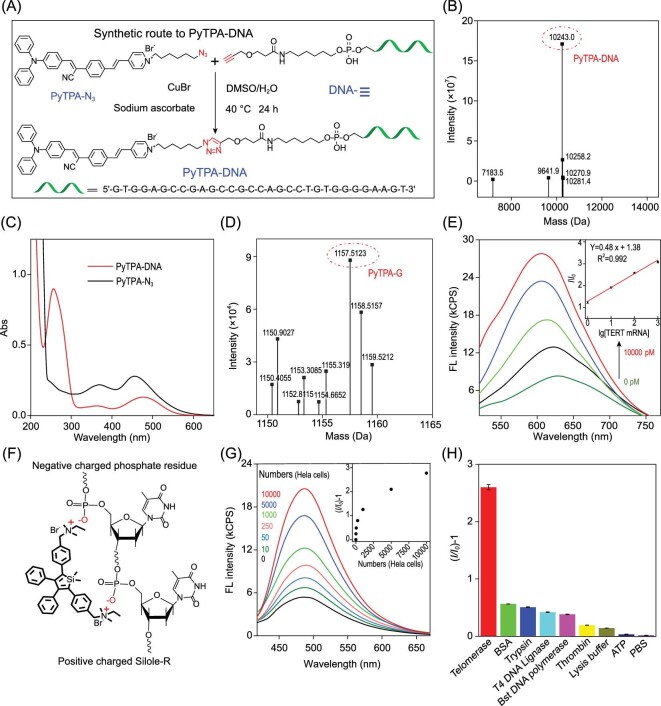
Feasibility analysis of PyTPA-DNA and Silole-R bioprobes in analyzing TERT mRNA and telomerase activity. (A) The synthetic route of PyTPA-DNA. (B) HRMS of PyTPA-DNA. (C) The UV–Vis spectra of PyTPA-N_3_ and PyTPA-DNA. (D) HRMS of the hydrolysis product PyTPA-G. (E) Fluorescence responses enhanced progressively with different concentrations of targets (0 pM, 10 pM, 100 pM, 1000 pM and 10 000 pM). (F) Electrostatic interaction between Silole-R and phosphate residues. (G) Fluorescence responses of Silole-R incubation with different concentrations of telomerase extracts. (H) Fluorescence responses (*I/I_0_*)−1 of telomerase versus different proteins under the same experimental conditions.

PyTPA-N_3_ was prepared according to the reported procedures [45] (Supplementary Fig. 1) and then coupled with Alk-DNA to yield PyTPA-DNA (Fig. 1A). The rude product was purified by High Performance Liquid Chromatography (HPLC) (Supplementary Fig. 1) and confirmed by High Resolution Mass Spectrometer (HRMS) with a peak at *m/z* 10 243.0 (Fig. 1B). The UV–Vis spectra of PyTPA-DNA exhibited a characteristic peak at 260 nm and 452 nm (Fig. 1C), respectively. The stability of PyTPA-DNA (Supplementary Fig. 2) in biological environments was confirmed. Nondenaturing polyacrylamide gel electrophoresis (PAGE) was used to demonstrate the stability of the PyTPA-DNA probes and analyze the target recycling process (Supplementary Figs 3 and 4) with assistance from Exo III. Furthermore, the mass spectrum of PyTPA-G (the hydrolysis product) was manifested at 1157.5 (Fig. 1D); the average hydrodynamic diameter of PyTPA-G was observed around 260 nm (Supplementary Fig. 5); the fluorescence intensity of PyTPA-DNA was significantly increased owing to S1 nuclease cleavage to form aggregated PyTPA-G in solution (Supplementary Fig. 6). Those results verified the successful synthesis of PyTPA-DNA and suggested that a target recycling strategy was an effective tool for detection.

On the other hand, the fluorescence intensity of PyTPA-DNA was gradually enhanced (2.9-fold) with the increased concentration of target from 0 to 10 000 pM and found to be linearly correlated with target concentrations from 10 to 10 000 pM, with detection limit of 3.36 pM (R^2 ^= 0.992) (Fig. 1E). Next, four different enzymes were used instead of Exo III in the reaction (Supplementary Fig. 7). As a result, almost no fluorescence was observed under the same treatment, suggestive of the important role of Exo III in the system. The fluorescence in response to perfectly matched strands was verified to be superior to the single/three base mismatch sequence (Supplementary Fig. 8). Finally, the fluorescence intensity of PyTPA-DNA incubated with different concentrations of RNA extracts was increased and represented reasonable correlation with different numbers of cells (Supplementary Fig. 9). Therefore, those results confirmed the effectiveness of the PyTPA-DNA in detecting TERT-mRNA.

The synthetic route of Silole-R was reported by our literature [32,33]. Based on electrostatic force, the Silole-R was able to interact with the negatively charged DNA backbone (Fig. 1F). The fluorescence of Silole-R was weak in the solution or incubating with short single-stranded DNA (Supplementary Table 2). While, in the presence of longer sequences (Ex-6, 54-nt), a rapid fluorescence was lighted by the aggregation state of Silole-R (Supplementary Fig. 10).

The fluorescent intensities of TP and Silole-R were enhanced gradually with HeLa cells rising from 0 to 10 000, which demonstrated the positive correlation between the fluorescence emission and the cell extracts (Fig. 1G, inset). However, HeLa cells pretreated with 100 *μ*M telomerase inhibition AZT (3^′^-azido-3^′^-deoxythymidine) exhibited no fluorescence (Supplementary Fig. 11). Only active telomerase, not other substrates, was able to initiate a potent fluorescence, suggestive of a high specificity of this bioprobe (Fig. 1H and Supplementary Fig. 12). Moreover, PAGE analysis and the TRAP method were employed to monitor the active telomerase assisted primer DNA extension, which indicated the successful elongation of DNA products with the help of telomerase (Supplementary Figs 13 and 14).

### Cell-cycle-regulated TERT mRNA and telomerase activity

**Figure 2.**
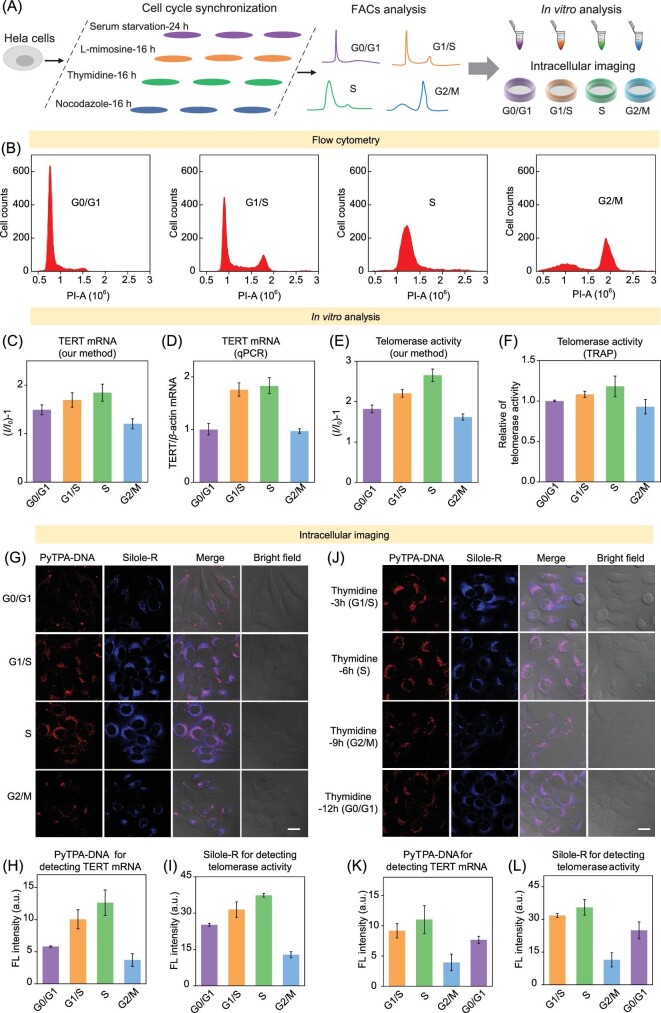
(A) Illustration of brief experimental procedure. (B) Cells were synchronized at the phases of G0/G1, G1/S, S and G2/M. (C–D) Quantification of TERT mRNA in HeLa cells by PyTPA-DNA bioprobe (*I*, average fluorescence intensity at 650 nm of cancer cell samples; *I_0_* is the blank samples) and qPCR. (E–F) Quantification of telomerase activity in HeLa cells with Silole-R bioprobe (*I*, average fluorescence intensity at 478 nm of cancer cell samples; *I_0_* is the blank samples) and TRAP method. (G–I) Images of TERT mRNA and telomerase activity in different HeLa cells synchronized at the G0/G1, G1/S, S and G2/M phases and their corresponding fluorescence intensity. (J–L) Images of TERT mRNA and telomerase activity in different HeLa cells synchronized at the G1/S, S, G2/M and G0/G1 phases and their corresponding fluorescence intensity. All scale bars are 20 *μ*m.

Four stages of HeLa cells were obtained by treatment of serum starvation, L-mimosine, thymidine and nocodazole respectively (Fig. 2A). Corresponding synchronized cell fractions were identified by flow cytometry analysis according to the DNA content (Fig. 2B and Supplementary Fig. 15).

First, PyTPA-DNA and Silole-R bioprobe were used to investigate the expression of TERT mRNA and telomerase activity under different cell cycles of HeLa cells. Figure 2C compares the fluorescence intensities (Supplementary Fig. 16) in different cell cycles. Upon progression through the cell cycle, the PyTPA-DNA faintly fluoresced in the G0/G1 stage but demonstrated an enhancement of fluorescence in response to the G1/S phase, and finally reached the strongest output in S stage. However, cells arrested at the G2/M phase showed the weakest fluorescence in contrast to the other three cell cycles. Furthermore, cell-cycle-dependent alterations of TERT mRNA expression in HeLa cells were reconfirmed by qPCR (Fig. 2D). The similar fluorescence responses of Silole-R lighted by active telomerase during different cell cycles were also received (Fig. 2E and Supplementary Fig. 17). Moreover, TRAP assay was selectively analyzed and found to have the strongest expression levels in S stage (Fig. 2F and Supplementary Fig. 18). Those detecting results revealed that the TERT mRNA amount and telomerase activity from different cancer cell cycle extracts were cell cycle dependent.

Second, the intracellular imaging of TERT mRNA and telomerase activity during different stages of cancer cell cycle were carried out. With minimal cytotoxicity of PyTPA-DNA and Exo III toward cells (Supplementary Fig. 19), PyTPA-DNA lit up HeLa cells by distributing in the cytoplasm and gradually boosted with high intensity for 2 h (Supplementary Fig. 20). Additionally, epigallocatechin gallate (EGCG) was utilized for dose-dependent inhibition of TERT mRNA expression. When incubated with HeLa cells at a concentration of EGCG of 250 *μ*g/mL, no fluorescence was observed, implying an inhibition on TERT mRNA expression in HeLa cells and the excellent biocompatibility of PyTPA-DNA probe (Supplementary Fig. 21). Silole-R and TP were used to monitor the cellular fluorescence responses with AZT or EGCG-treatment (Supplementary Figs 22 and 23). The fluorescence of Silole-R was lighted up when coexisting with HeLa cells without any treatment. Furthermore, the intracellular fluorescence results demonstrated that the bioprobes could successfully monitor the intracellular TERT mRNA level and telomerase activity when coexisting with HeLa cells (Supplementary Fig. 24). As such, because of the fluorescence switch-on visualization of TERT mRNA and telomerase activity in cells, imaging capabilities of high accuracy, sensitivity, and positive relationship between fluorescence intensity and TERT mRNA or telomerase activity were achieved by our system.

Fluorescence imaging of the expression of TERT mRNA (Supplementary Fig. 25) and telomerase activity (Supplementary Fig. 26) were carried out in different synchronized HeLa cells. After incubating a cell with two probes, an enhancement of the cellular fluorescence intensity was observed upon most cells; both red fluorescence and blue fluorescence were gradually increased in G1/S and S phase (Fig. 2G), potentially owing to increasing activity of telomerase over cell division from G0/G1 to S phase. Furthermore, the average fluorescence drastically reduced in G2/M phase cells, which was almost half of the initial fluorescence. The intracellular fluorescence intensities of PyTPA-DNA corrected against background signal in the cell cycle of G0/G1 phase (6.8 a.u.), S phase (10.1 a.u.) and G2/M phase (3.7 a.u.) are shown in Fig. 2H. The fluorescence intensity of Silole-R corrected against background signal for HeLa cells resident in G0/G1, S and G2/M phase of the cell cycle accounted for 16.7 a.u., 48.5 a.u. and 32.7 a.u., respectively (Fig. 2I). To further confirm the contribution of the cell cycle to varied responses of fluorescence, cellular fluorescence was selectively analyzed when treated with thymidine, which was reported to affect cell behaviors to obtain different cell cycle [46,47]. Thus, fluorescence of HeLa cells treated with thymidine was monitored and found to demonstrate the highest value in S phase and related lower value in G2/M phase and almost the same signal in G1/S and G0/G1 stages (Fig. 2J–L). The intracellular fluorescence imaging results demonstrated that the cell cycle has dramatic effects on the localization of TERT mRNA and telomerase activity—the telomerase was specific boosted in the S phase and reduced in the G2/M phase in human cancer cells.

### Cell-cycle-regulated TERT mRNA and telomerase activity in clinical tissue samples

**Figure 3.**
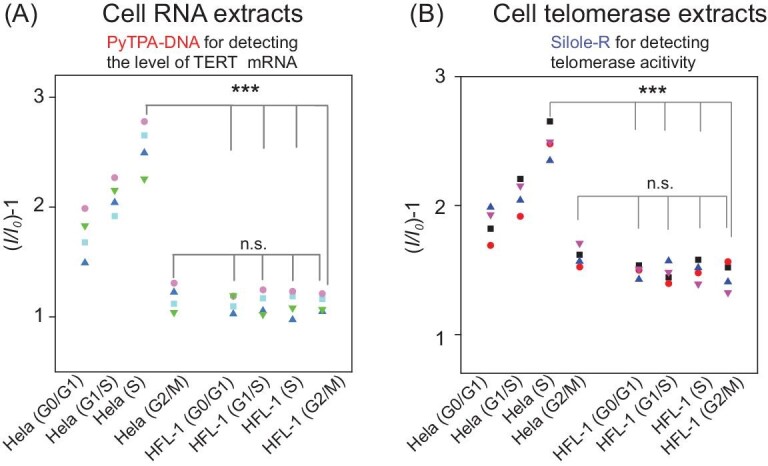
(A) Fluorescence response *I/I_0_* of TERT mRNA in different phases of HeLa cells and normal cells (*I*, average fluorescence intensity at 650 nm of cancer cell samples; *I_0_*, blank samples). (B) Fluorescence response *I/I_0_* of telomerase activity in different phases of HeLa cells and normal cells (*I*, average fluorescence intensity at 478 nm of cancer cell samples; *I_0_*, blank samples).

We tested the expression level of TERT mRNA and telomerase activity in different periods of HeLa cells, human lung fibroblasts (HFL-1) cells and tissue specimens. Serum starvation at different times induced HFL-1 cells to arrest at different phases of the cell cycle (G0/G1, G1/S, S and G2/M) [48]. The expression level of TERT mRNA and active telomerase in normal cells revealed that somatic cells have almost no activation of telomerase during three phases of the cell cycle (Fig. 3A and B). The levels of TERT mRNA and active telomerase in most phases of the cell cycle (G0/G1, G1/S, S) are above normal cells according to the definition of (*I/I_0_*) − 1, while cells arrested in G2/M phase exhibited almost the same level of HFL-1 as normal cells. Those results remind us to take cell cycle into consideration when we use TERT mRNA and telomerase activity as the early diagnosis of tumor lesion.

**Figure 4.**
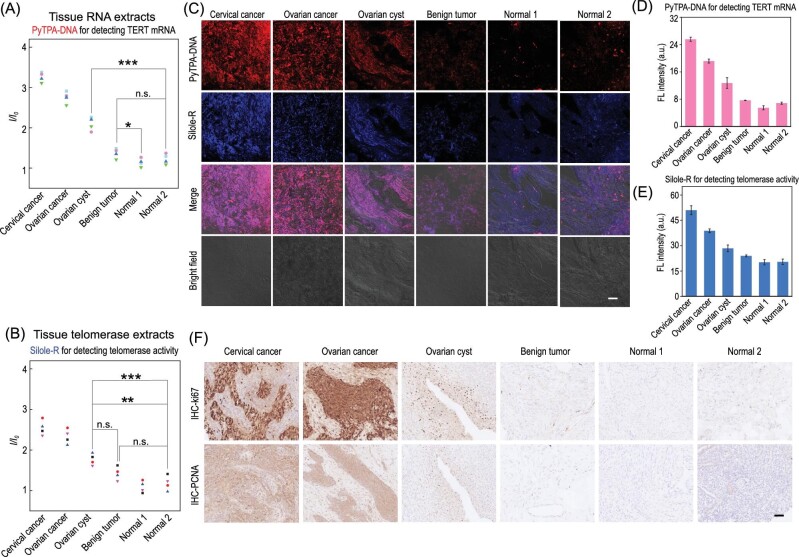
(A and B) Fluorescence response *I/I_0_* of TERT mRNA and telomerase activity in various tissue specimens. (C–E) Images of the expression of TERT mRNA, telomerase activity in different tissue specimens and their corresponding fluorescence intensities. (F) The IHC staining images of Ki67 and PCNA in tissue specimens. Scale bar, 20 *μ*m.

To verify the application value of our proposed imaging strategy in clinical samples, both PyTPA-DNA and Silole-R bioprobes were employed to detect the level of TERT mRNA and active telomerase in tissue specimens from clinical patients, such as malignant tumor, ovarian cyst, benign tumor and normal samples. The detecting results of TERT mRNA and telomerase activity showed signal increase ratios of 2/2 (100%) for cancer tissues and 1/2 (50%) for ovarian cysts from normal tissues (Fig. 4A and B) according to the definition of fluorescence intensity ratios between the normal specimens.

We further examined the expressions of TERT mRNA and active telomerase in tissues by using our proposed imaging strategy. As shown in Fig. 4C–E, Confocal Laser Scanning Microscope (CLSM) images showed that two cancer tissues displayed obvious red and blue fluorescence intensity when incubating with PyTPA-DNA and Silole-R, respectively. The tissue of cervical cancer and ovarian cancer was mainly located in the stage of cell proliferation, which was verified by immunohistochemical (IHC) staining for Ki67 and Proliferating Cell Nuclear Antigen (PCNA) (Fig. 4F). Moreover, fluorescence intensity of ovarian cysts was much higher than that of normal tissues. Both normal tissues exhibited almost non-fluorescence in the presence of PyTPA-DNA and Silole-R. On the other hand, the tissues were consistent with hematoxylin-eosin (H&E) staining (Supplementary Fig. 27).

### Other biomarkers also vary as the cell cycle progresses

**Figure 5.**
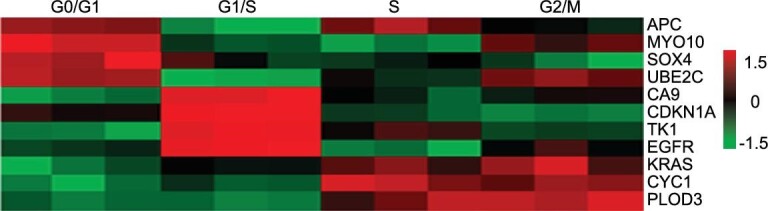
The expression of tumor markers during different phases of the cell cycle.

In order to acquire a map of transcriptome information during different phases of the cell cycle, RNA sequencing was performed by using dividing cells synchronization. First, a list of known cell-cycle-regulated genes was selected [49,50] and found to have peak expression in each of our groups (Supplementary Fig. 28). Then, the level of TERT gene expression in each cell cycle was investigated (Supplementary Fig. 29) and it was consistent with the above-mentioned results. Next, great interest is aroused in analyzing the expression of genes and

corresponding pathways during different phases of the cell cycle (Supplementary Figs 30–35), which is an important research area related to disease screening. Moreover, some biomarkers for various cancer detection were casually chosen to determine their expression level during different phases of the cell cycle, which suggested that tumor biomarkers were highly diversified. As shown in Fig. 5, the parameters of CA9, CDKN1A, TK1 and EGFR were significantly elevated in G1/S stage. However, the activities of KRAS, CYC1 and PLOD3 were remarkably weakened in G0/G1 and G1/S phases. Based on evaluation, these results indicated that different tumor markers were highly diversified and varied in the functions of different cell cycles.

## CONCLUSION

In summary, we have constructed a highly sensitive system for imaging the cellular expression of TERT mRNA and telomerase activity during different phases of the cell cycle based on AIEgens. We found that TERT mRNA and telomerase activity varied during the cell cycle and were able to affect the accuracy of cancer identification. The fluorescence intensities of cancer cells arrested at the phases G1/S and S were increased. In contrast, the cancer cells arrested at the phase G2/M and normal cells showed a negligible fluorescence intensity, which suggested a significant role of the cell cycle progression in regulation of telomerase activation. Herein, the cell cycle accounted for the sensitivity and efficiency of diagnosis in the field of telomerase identification.

It is worth noting that the cell cycle had a major role in cellular processes and had the ability to modulate various biomarkers. Some tumor biomarkers varied as the cell cycle progressed, such as CA9, TK1 and EGFR, which were more abundantly expressed at early S stage. Hence, our AIEgens bioprobes provided potent tools of differentiation of TERT mRNA and telomerase activity in the cell cycle and an important guidance tool for the development of probes for cell-cycle-dependent detection.

## METHODS

### Telomerase activity detection by Silole-R bioprobe

Telomerase extracts from different numbers of HeLa cells and HFL-1 cells were first diluted in lysis buffer and stored in the −80°C refrigerator. Telomerase extracts and 7.8 *μ*M Silole-R with dNTPs, TP and RNase inhibitor were incubated at 37°C for 1 h, then transferred to 94°C for 5 min to end the extension. Finally, the fluorescence emission spectra were measured in the range from 400 to 700 nm (λ_ex_= 360 nm).

### TERT mRNA detection by PyTPA-DNA

The 50 *μ*L solution consisted of PyTPA-DNA (10 *μ*mol/L), Exo III (1 U/*μ*L), Helps 1-2 (4 *μ*mol/L), dNTPs (200 *μ*mol/L), recombinant RNase inhibitor (0.8 U/*μ*L) and varying concentrations of RNA extracts. After incubation at 37°C for 1 h, the reaction mixture was transferred to 95°C for 5 min to stop the reaction.

### Electrophoresis experiment

PAGE (12%) in 1 × TBE was used to test different samples (with 1 × loading buffer) at 100 V, 24°C. After separation, the gel was stained with Gel Red and photographed under a UV lamp.

### Cellular imaging of TERT mRNA and telomerase activity

HeLa cells were seeded in a 20 mm confocal dish for 24–48 h. For imaging of TERT mRNA, PyTPA-DNA (5 *μ*M) and 0.5 U/*μ*L Exo III were transfected into cells by using lipofectamine 2000 (4 *μ*L) in Opti-MEM for 1–3 h. Subsequently, transfection mixtures were removed from cells by using PBS buffer. For imaging of telomerase activity, the 3.6 *μ*M TP was transfected using 3 *μ*L of lipofectamine-2000 in Opti-MEM at 37°C for 1 h. Subsequently, 5.0 *μ*M Silole-R was added into the medium for 30 min. Before the imaging observation, cells had to be washed three times by using PBS buffer.

### Clinical sample imaging of TERT mRNA

The clinical tissue samples were incubated with the above cellular TERT mRNA and telomerase activity imaging mixture for 1 h. Then, corresponding samples were washed with PBS buffer three times under a Zeiss LSM 880 confocal laser scanning microscope.

## Supplementary Material

nwaa306_Supplemental_File
