# Supplementary material for: Aggregation-induced emission luminogens reveal cell cycle-dependent telomerase activity in cancer cells
Source: Natl Sci Rev. 2021 Jan 2;8(6):nwaa306. doi: 10.1093/nsr/nwaa306 (PMC8288165; doi:10.1093/nsr/nwaa306)
Supplement: nwaa306_Supplemental_File [file nwaa306_supplemental_file.pdf]

Role of cell cycle progression on analyzing telomerase in cancer cells based on aggregation-induced emission luminogens

Xia Wu[1], Jun Wu[1], Jun Dai[2], Biao Chen[2], Zhe Chen[2], Shixuan Wang[2], Feng Wu[1], Xiaoding Lou[1,*] and Fan Xia[1]

[1] Engineering Research Center of Nano-Geomaterials of Ministry of Education, Faculty of Materials Science and Chemistry, China University of Geosciences, Wuhan 430074, China

[2] Department of Obstetrics and Gynecology, Tongji Hospital, Tongji Medical College, Huazhong University of Science and Technology, Wuhan 430074, China

*Corresponding authors. E-mails: louxiaoding@cug.edu.cn

Additional methods

Additional Data

Table of contents

Supplementary Tab. 1. DNA sequences designed in PyTPA-DNA probe for TERT mRNA detection. 7

Supplementary Tab. 2. DNA sequences designed in TP and Silole-R for telomerase activity detection. 7

Supplementary Fig. 1. The structure of molecule PyTPA-N_3.. 8

Supplementary Fig. 2. The stability of PyTPA-DNA. 9

Supplementary Fig. 3. PAGE analysis. 10

Supplementary Fig. 4. The enzymatic digestion process and the structure of PyTPA-G. 10

Supplementary Fig. 5. Dynamic light scattering measurements. 11

Supplementary Fig. 6. The fluorescence intensity of the PyTPA-DNA in the presence of S1 nuclease. 11

Supplementary Fig. 7. The relative fluorescence intensity of proteins in stead of Exo III. 12

Supplementary Fig. 8. Fluorescence response of PyTPA-DNA toward PM, SM and TM. 12

Supplementary Fig. 9. Fluorescence intensity of PyTPA-DNA probes with RNA extracts. 13

Supplementary Fig. 10. Fluorescence emission spectra of Silole-R with different oligonucleotides. 13

Supplementary Fig. 11. Fluorescence emission spectra of telomerase extracted from AZT treated HeLa cells. ... 14

Supplementary Fig. 12. Fluorescence intensity of proteins used in place of telomerase to test the specificity. 15

Supplementary Fig. 13. Non-denaturating PAGE analysis of telomerase extension assay. 16

Supplementary Fig. 14. Representative TRAP gel electrophoresis of telomerase products. 16

Supplementary Fig. 15. Percentage of cells in individual cycle phases after different treatments. 17

Supplementary Fig. 16. Fluorescence spectra of PyTPA-DNA with RNA extract from different HeLa cells 17

Supplementary Fig. 17. Fluorescence spectra of Silole-R in the presence of different HeLa cells 18

Supplementary Fig. 18. Telomerase activity from different HeLa cells and tested by quantitative TRAP assays. 18

Supplementary Fig. 19. The cytotoxicity of PyTPA-DNA was tested with HeLa cells by MTT assay. 19

Supplementary Fig. 20. LSCM images of PyTPA-DNA in the presence of Exo III for different times. 20

Supplementary Fig. 21. LSCM images of HeLa cells with EGCG stimulation. 21

Supplementary Fig. 22. LSCM images of HeLa cells pretreated without or with AZT. 22

Supplementary Fig. 23. LSCM images of HeLa cells pretreated with different concentrations of EGCG. 23
Supplementary Fig. 24. Time course of LSCM images of HeLa cells arrested in different phases of cell cycle. .. 24
Supplementary Fig. 25. LSCM images of HeLa cells in different phasess with PyTPA-DNA. 25
Supplementary Fig. 26. LSCM images of HeLa cells with TP and Silole-R in different phases. 26
Supplementary Fig. 27. H&E staining of different tissues 27
Supplementary Fig. 28. Gene expression profiles of well-characterized cell cycle genes 28
Supplementary Fig. 29. The expression of TERT in different cell cycle clusters.. 28
Supplementary Fig. 30. Profiling of different expression genes in G0/G1 and G1/S phases of cell cycle............ 29
Supplementary Fig. 31. Profiling of different expression genes in G0/G1 and S phases of cell cycle.................. 30
Supplementary Fig. 32. Profiling of different expression genes in G0/G1 and G2/M phases of cell cycle 31
Supplementary Fig. 33. GO analysis of the BP terms in G1/S vs G0/G1 of HeLa cell lines. 32
Supplementary Fig. 34. GO analysis of the BP terms in S vs G0/G1 of HeLa cell lines. 33
Supplementary Fig. 35. GO analysis of the BP terms in G2/M vs G0/G1 of HeLa cell lines. 34

Additional methods

Cell culture and telomerase extracted

HeLa cells were cultured in DMEM (Gibco) medium supplemented with 10% FBS and 1% penicillin-streptomycin. HFL-1 cells were cultured in F-12 (Gibco) medium supplemented with 10% FBS and 1% penicillin-streptomycin at 37 °C in 5% CO_2. Telomerase extracted from cultured cells using the CHAPS method. Cell pellets (1 million cells) were first suspended in 200 μL 1x CHAPS lysis buffer and incubated on ice for 30 min. Then, the mixture was spun at 12,000 g for 20 min at 4 °C, the supernatant was partial shipments into a fresh tube and stored at -80 °C until use.

Telomerase extracted from tissues

Telomerase extracted from tissues using the repeated freezing and thawing of liquid nitrogen to get the single cells, the next steps were as same as telomerase extracted from cultured cells.

RNA extracted from cells and tissues

HeLa cell pellets were seeded at 1×10^5 mL^{-1} in DMEM with different kinds of treatment, then harvested and RNA were extracted following a regular Trizol method, according to the manufacturer's instructions. Tissues were extracted following a regular Trizol method, according to the manufacturer's instructions.

Cell cycle synchronizations

The synchronization of HeLa cells was adapted from the protocol as described [1,2]. To synchronize at G0/G1 phase, HeLa cells were arrested by serum starvation. The exponentially growing cells (1.0×10^6 cells) were plated onto dishes with DMEM without FBS, and incubated for 24 h before

harvesting. To synchronize at G1/S phase, HeLa cells were plated in 9 cm dishes in complete media and allowed to attach for 16 h, reaching 70% confluence. Then, cells were arrested by L-mimosine for 16 h before harvesting. To synchronize at S phase, HeLa cells were plated in 9 cm dishes in complete media and allowed to attach for 16 h, reaching 70% confluence. Then, cells were incubated in DMEM containing 2 mM thymidine for 16 h before harvesting. To synchronize at G2/M phase, HeLa cells were plated in 9 cm dishes in complete media and allowed to attach for 16 h, reaching 80% confluence. Then cells were arrested by complete media containing 30 ng/mL nocodazole for 16 h before harvesting. HFL-1 cells were subjected to serum deprivation for 24 h and thereafter resupplied with serum. After starvation, the cells were passaged and released into cell cycle by addition of serum. Re-feeding with serum resulted in a clear enrichment of G1/S (16 h), S (20 h) and G2/M (24 h) phase in HFL-1 cells.

Flow cytometric analysis

The extent of synchronization was measured using flow cytometry as follows; the attached cells (1.0×10^6 cells) were first trypsinized, centrifuged at 100×g for 5 min, and then fixed in 75% ethanol at 4 °C. After overnight fixation, cells were centrifuged at 100×g for 5 min, followed by resuspending in PBS containing 2 mM EDTA and RNase A (0.2 mg/ml, Sigma), and incubated for 10 min at 37 °C. Propidium iodide (0.02 mg/ml, examine DNA content) was then added and incubated for 20 min. The synchronization of the cells was evaluated using a flow cytometric analysis (ACEA-NovoCyte 2040R), and results were analyzed with the software of NovoExpress 1.3.0.

Quantitative real-time PCR analysis

Quantitative real time PCR analysis was obtained by using the Power SYBR Green PCR Master kit (Applied Biosystem). A total of 1 μg RNA of each sample was used for RT-PCR. cDNA was synthesized by reverse transcription as described by the manufacturer and amplified in a 20 μL of reaction system containing 10 μL of SYBR premix, 0.4 μL of each primer, 0.4 μL of ROX reference Dye II and 0.1 μL of cDNA template at the following reaction conditions: initial denaturation at 95 °C for 5 min followed by 40 cycles of 95 °C for 10 s, 60 °C for 30 s. The primers used for the PCR amplifications are as follows: TERT gene (forward, 5′- ATGTCACGGAGACCACGTTT-3′; reverse, 5′-GCACCCTCTTCAAGTGCTGT-3′) and β-actin gene (forward, 5′-GTCCACCGC AAATGCTTCTA-3′; reverse, 5′-TGCTGTCACCTTCACCGTTC-3′). The melting curve of PCR

was analyzed. The experiment was repeated three times and β-actin was used as an internal reference. Relative expression of TERT to β-actin was calculated using $2^{-\triangle\triangle CT}$.

Telomeric Repeat Amplification Protocol (TRAP) assay

Telomerase activity in HeLa cells was used as described previously with a slight modification. A 0.5 μL portion of the test solution was added to a mixture of 8.3 μL of TRAP buffer solution and 0.5 μL of the cell extract, and the mixture was pre-incubated on ice for 20 min. After addition 0.7 μL of a solution that contained 25 ng of telomerase primer (5'-AATCCGTCGAGCAGAGTT-3') and 0.5 nmol of dNTP mixture, the reaction mixture was incubated at 37 °C for 60 min and then heated at 95 °C for 5 min in order to inactivate telomerase. After the reaction mixture was cooled to room temperature, additions of 0.2 U of Taq polymerase and 5 μL of dNTP solution (5 mM), 2.59 μL of 1 μg/μL ACX primer solution (5'-GCGCGGCTTACCCTTACCCTTACCCTAACC-3'); PCR was then conducted for 30 cycles (94 °C for 30 s, 58 °C for 30 s, and 72 °C for 30 s). The PCR products were analyzed by 12% polyacrylamide gel PAGE in TBE buffer at 100 V for 2 h, then stained with Gel Red and photographed under a UV lamp.

Cytotoxicity assay

The cytotoxicity of PyTPA-DNA and EXO III on HeLa cells were assessed using standard MTT assays. First, 5×10^3 cells/mL cells were seeded onto 96-well plates and grown for 24 h. The cells were treated with various concentrations of PyTPA-DNA (0-10 μM) and EXO III (0-2 U/μL) for 24 h. After that, a total 10 μL of MTT solution (5 mg/mL) was added into every well incubated at 37 °C for 4 h. Finally, the supernatants were aspirated and then 150 μL of DMSO was added to each well. The absorbance values of the wells at 540/570 nm was performed on a microplate reader.

In situ imaging of TERT mRNA and telomerase activity

All cellular fluorescent images were collected with biological confocal laser scanning microscope (model FV1200). A 488 nm laser was used for the excitation of PyTPA-DNA, and the fluorescence emission was collected at 580-700 nm. A 405 nm laser was used for the excitation of Silole-R, and the fluorescence emissions were collected at 400-500 nm. The images were acquired using a 60×oil immersion objective lens. The fluorescence intensity values in the LSCM imaging assay were analyzed by quantifying the average fluorescence intensity of LSCM images with the image processing software.

H&E staining and immunohistochemical (IHC) Staining.

H&E staining was conducted according to the manufacturer's instructions. Specimens were fixed in 10% neutral-buffered formalin, dehydrated in graded alcohols, and then embedded in paraffin. Sections (1 mm) were cut and then stained with hematoxylin and eosin (H&E). IHC staining was performed with reference to our previous studies. Briefly, the obtained tumor tissues were cut into 4 μm-thick sections for IHC staining. After deparaffinization, antigen retrieval, and BSA blocking, the tissue sections were incubated with primary antibodies (Ki67 and PCNA) at 4 °C overnight, followed by washing 3 times with PBS. Then the secondary antibody (DAKO) was added dropwise, and incubation was continued for 1 h at 37 °C. The tissue sections were washed with PBS for 3 times again. Finally, the sections were stained using 3, 3′-diaminobenzidine, counterstained with hematoxylin, and observed as well as recorded under a microscope.

Transcriptome analysis

cDNA synthesis and cDNA library construction

Total RNA was isolated using the Trizol Reagent (Invitrogen Life Technologies), after which the concentration, quality and integrity were determined using a Nanodrop spectrophotometer (Thermo Scientific). Three micrograms of RNA were used as input material for the RNA sample preparations. Sequencing libraries were generated using the TruSeq RNA Sample Preparation Kit (Illumina, San Diego, CA, USA). Briefly, mRNA was purified from total RNA using poly-T oligo-attached magnetic beads. Fragmentation was carried out using divalent cations under elevated temperature in an Illumina proprietary fragmentation buffer. After adenylation of the 3′ ends of the DNA fragments, illumina PE adapter oligonucleotides were ligated to prepare for hybridization. To select cDNA fragments of the preferred 380 bp in length, the library fragments were purified using the AMPure XP system (Beckman Coulter, Beverly, CA, USA). DNA fragments with ligated adaptor molecules on both ends were selectively enriched using illumina PCR Primer Cocktail in a 15 cycle PCR reaction. Products were purified (AMPure XP system) and quantified using the Agilent high sensitivity DNA assay on a Bioanalyzer 2100 system (Agilent). The sequencing library was then sequenced on a Hiseq X ten platform/NovaSeq 6000 (Illumina) by Shanghai Personal Biotechnology Cp. Ltd.

Basic analysis ： Samples are sequenced on the platform to get image files, which are transformed by the software of the sequencing platform, and the original data in FASTQ format (Raw Data) is generated. Sequencing data contains a number of connectors, low-quality Reads, so we use Cutadapt

(v2.7) [3] software to filter the sequencing data to get high quality sequence (Clean Data) for further analysis. In data mapping analysis, the reference genome and gene annotation files were downloaded from genome website. Reference genome index was built by Bowtie2 (v2.4.1) [4] and the filtered reads were mapping to the reference genome using HISAT2 (2.1.0) [5], the default mismatch was no more than 2. The alignment region distribution of mapped reads was calculated.

mRNA analysis ： Firstly, we used HTSeq (0.11.1) [6] statistics to compare the Read Count values on each gene as the original expression of the gene, and then used FPKM to standardize the expression. Then we used DESeq (1.39.0) [7] to analyze the genes of difference expression with screened conditions as follows: expression difference multiple |log2FoldChange| > 1, significant P-value < 0.05. At the same time, we used R language Pheatmap (1.0.12) software package to perform bi-directional clustering analysis of all different genes of samples. We get heatmap according to the expression level of the same gene in different samples and the expression patterns of different genes in the same sample with Euclidean method to calculate the distance and Complete Linkage method to cluster. Next, we used topGO (2.40.0) to map all the genes to Terms in the Gene Ontology database and calculated the numbers of differentially enriched genes in each Term. Based on the whole genome, Terms with significant enrichment of differentially enriched genes were calculated by hypergeometric distribution. The purpose of GO enrichment analysis is to obtain GO functional terms with significant enrichment of differentially expressed genes, thus revealing the possible functions of differentially expressed gene in the samples, as well as we counted the number of differentially expressed genes at different levels of KEGG Pathway, and then determined the metabolic pathways and signaling pathways that differentially expressed genes mainly participate in.

Supplementary Tab.1. DNA sequences designed in TERT mRNA detection.

Name	Sequence (5' to 3')
**PM Target** (perfectly matched)	ACTTC CCCAC AGGCT GGCGC TCGGC TCCAC CCCAG GGCCA
**PyTPA-DNA**	PyTPA-GTGGA GCCGA GCGCC AGCCT GTGGG GAAGT
**SM DNA** (single base mismatch)	ACTTC C**T**CAC AGGCT GGCGC TCGGC TCCAC CCCAG GGCCA
**TM DNA** (three bases mismatch)	ACTTC C**T**CAC AGGCT GGC**A**C TCGGC T**G**CAC CCCAG GGCCA
**Help 1**	GGACTATTCCTATGTGGGGAGTGGAAGCCGGGC TCCTGGTGAGGAAAAGCTGGCCCTGGG
**Help 2**	GAAGACGGCAGGTGTGCTGGACACTCAGCCCTT GGCTGGACACTCGCTCAGGCCTCAGCC

Supplementary Tab. 2. DNA sequences designed for detecting telomerase activity.

Name	Sequence(5' to 3')	Length (nt)
**TP**	AATCCG TCGAGC AGAGTT	18
**Ex-0**	CCGAAT AGCTCG GTTAGA	18
**Ex-1**	CCGAAT AGCTCG GTTAGA **TTAGGG**	24
**Ex-2**	CCGAAT AGCTCG GTTAGA **TTAGGG** **TTAGGG**	30
**Ex-4**	CCGAAT AGCTCG GTTAGA **TTAGGG** **TTAGGG** **TTAGGG** **TTAGGG**	42
**Ex-6**	CCGAAT AGCTCG GTTAGA **TTAGGG** **TTAGGG** **TTAGGG** **TTAGGG** **TTAGGG** **TTAGGG**	54

Supplementary Fig. 1. (A) The structure of molecule PyTPA-N_3. (B) HRMS (MALDI-TOF) of compound PyTPA-N_3. (C) High performance liquid chromatography (HPLC) result of PyTPA-N_3. (D) The HPLC result of PyTPA-DNA.

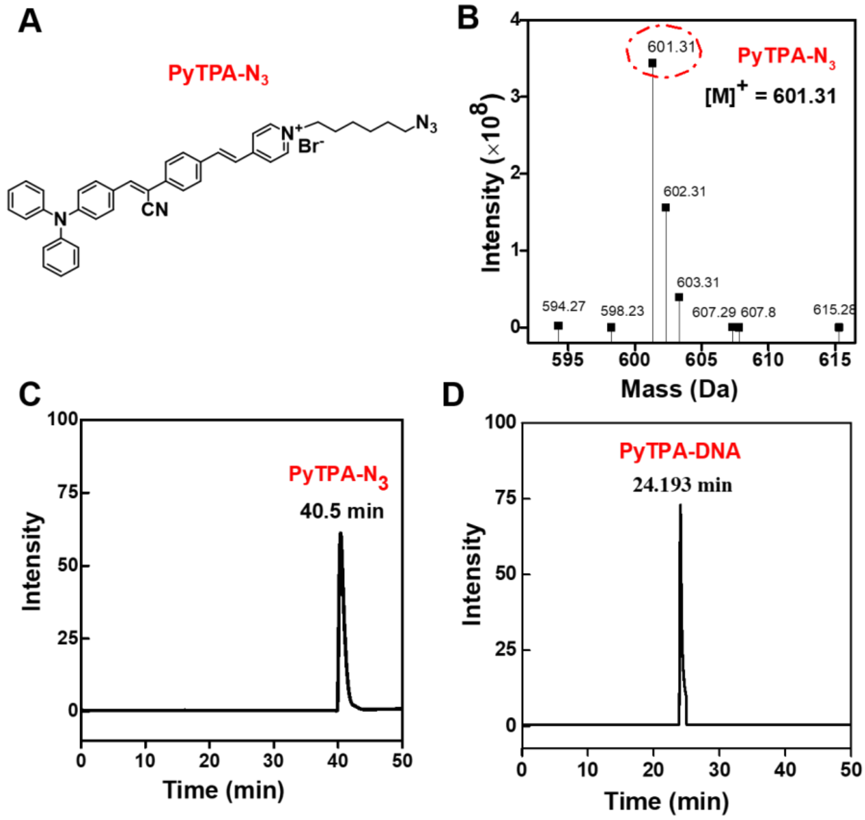

Supplementary Fig. 2. (A-F) The absorbance of PyTPA-DNA in the presence of pH 4, pH 8, pancreatic enzyme (10 %), 1 mM NaCl, cell lysates and serum for 0 h and 1 h. **(G-L)** The relative fluorescence intensity of PyTPA-DNA in the presence of 20 mM PBS (pH 4, 8), pancreatic enzyme (10 %), 1 mM NaCl, cell lysates and serum for 0 h vs 1 h.

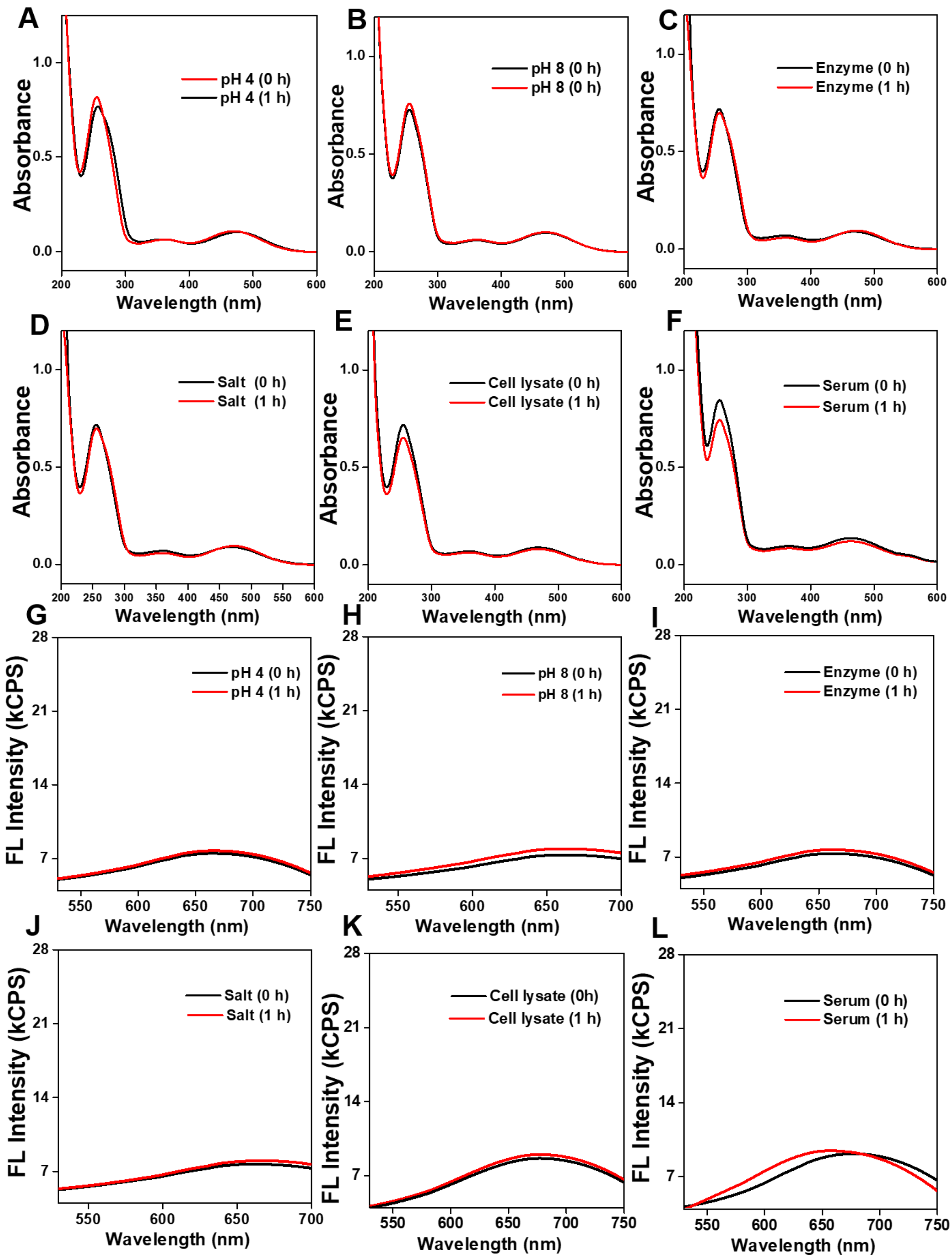

Supplementary Fig. 3. PAGE analysis was used to analyze the target recycling process with an assistance of exonuclease III (Exo III). A duplex structure was formed by the hybridization between PyTPA-DNA and PM target, while in the presence of Exo III, PyTPA-DNA was digested and the duplex was weakened to release PM target for next new cycle (lane 6), suggesting that target recycling strategy was an effective tool for mRNA detection. Lane 1, DNA Ladder; Lane 2, Alk-DNA; Lane 3, PyTPA-DNA probe; Lane 4, PM target DNA ; Lane 5, PM target DNA + PyTPA-DNA; Lane 6, PM target DNA + PyTPA-DNA + Exo III.

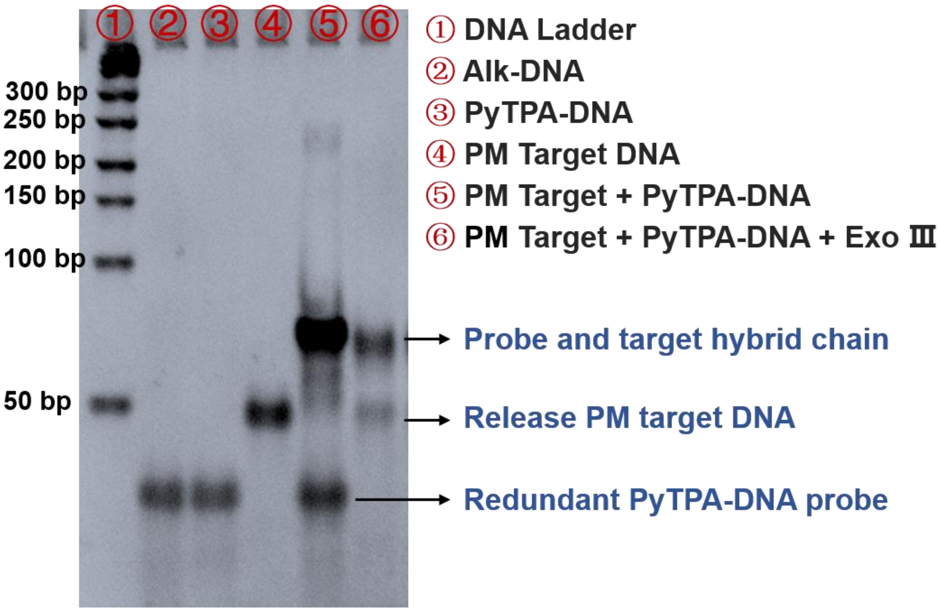

Supplementary Fig. 4. The enzymatic digestion process and the structure of product (PyTPA-G).

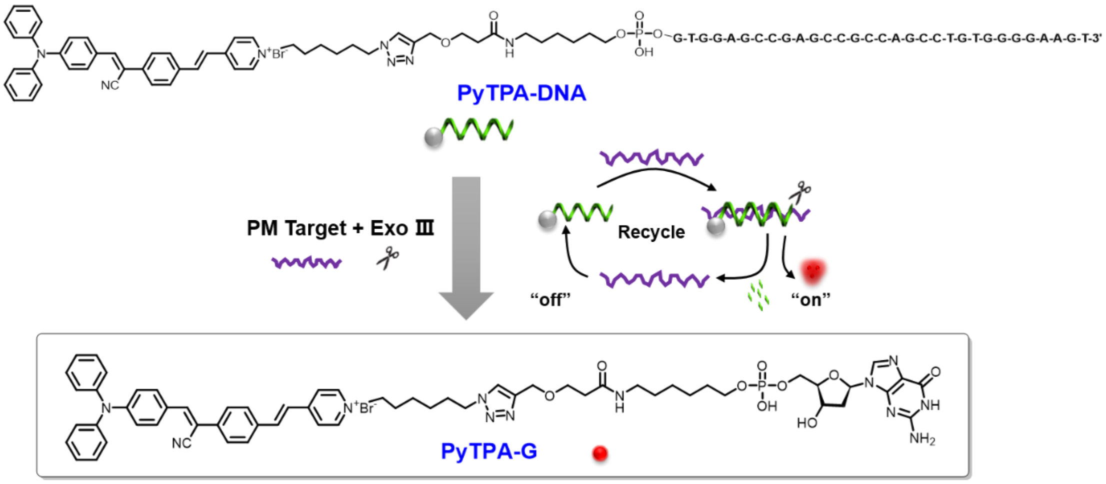

Supplementary Fig. 5. Dynamic light scattering measurements of PyTPA-DNA (red) and PyTPA-DNA incubated with mRNA and Exo III (green).

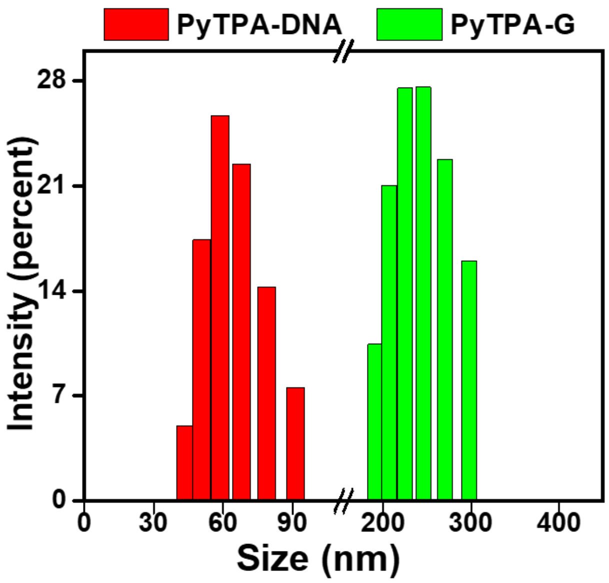

Supplementary Fig. 6. The fluorescence intensity of the PyTPA-DNA in the presence of S1 nuclease (10 U) is 375% higher than that in the absence of it.

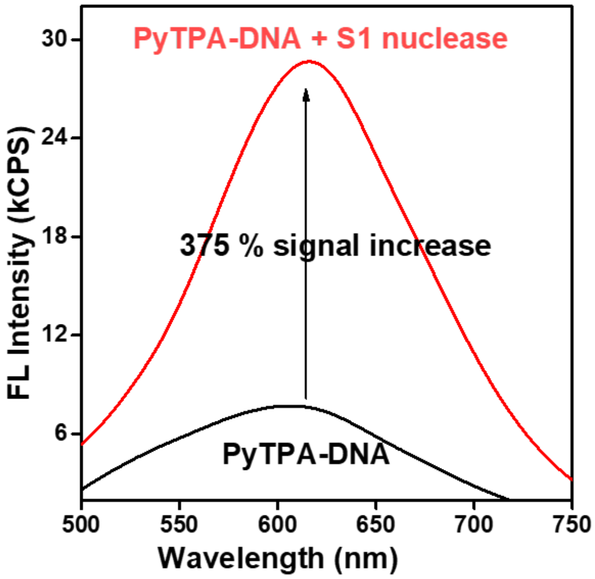

Supplementary Fig. 7. The relative fluorescence intensity and fluorescence respond of PyTPA-DNA in BSA, T4 DNA Ligase, trypsin, thrombin and PBS instead of Exo III.

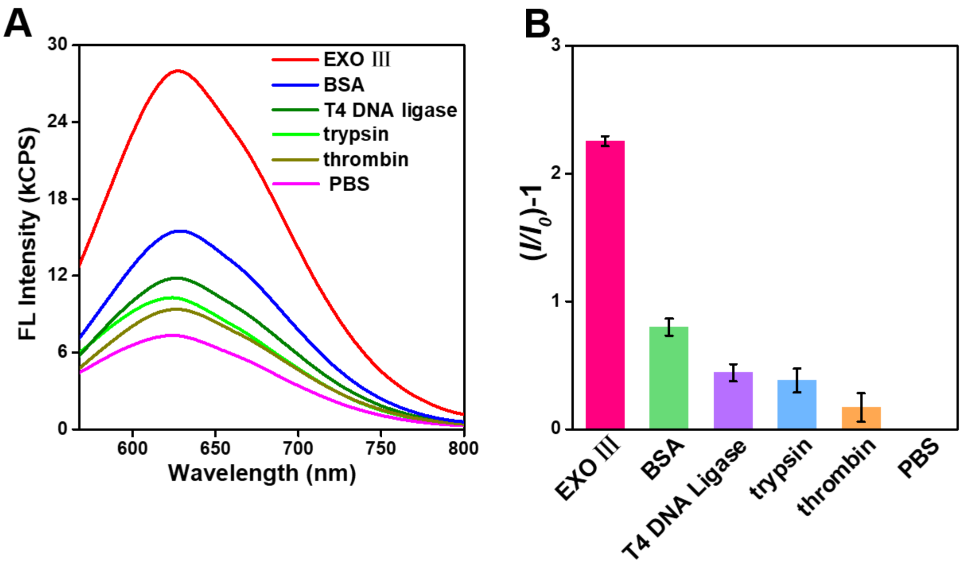

Supplementary Fig. 8. The relative fluorescence intensity and fluorescence response of PyTPA-DNA toward perfect match (PM), single mismatched targets (SM) and triple mismatched targets (TM). Specificity tests demonstrate that the assay could distinguish the perfect match (PM) targets with single/triple mismatched targets.

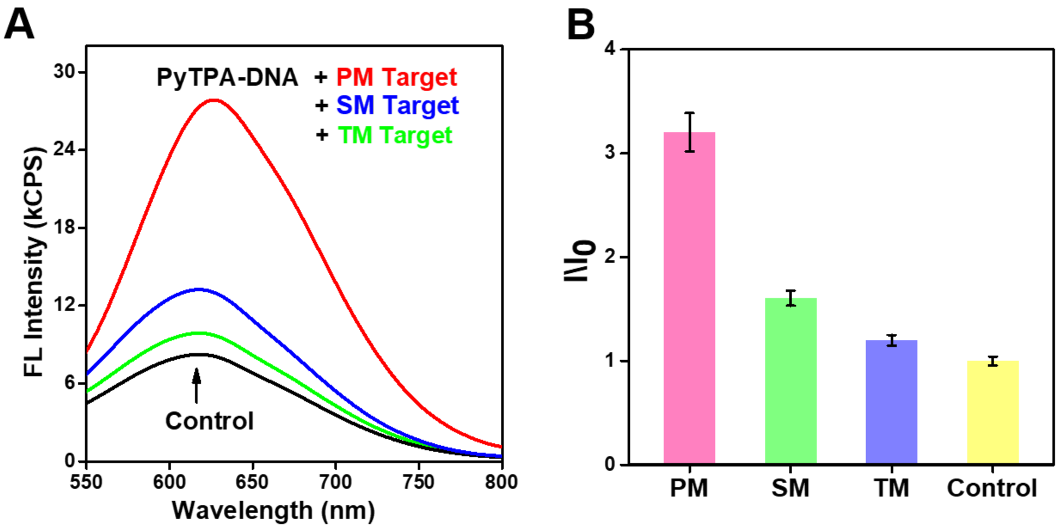

Supplementary Fig. 9. The relative fluorescence intensity and fluorescence response of PyTPA-DNA probes (10 μM) incubated without or with RNA extracts of 2000, 5000, 10000, 20000 HeLa cells.

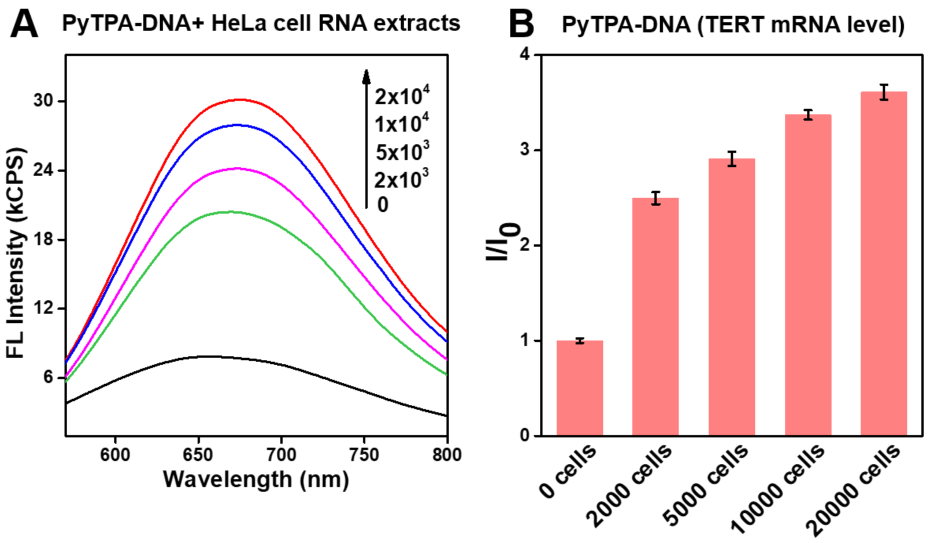

Supplementary Fig. 10. (A) Fluorescence emission spectra of Silole-R in the absence and presence of different oligonucleotides (Ex-0, Ex-1, Ex-2, Ex-4 and Ex-6). (B) Linear relationship between (I/I_0)-1 at 478 nm and the lengths of oligonucleotides. The concentration of Silole-R is 7.5 μM.

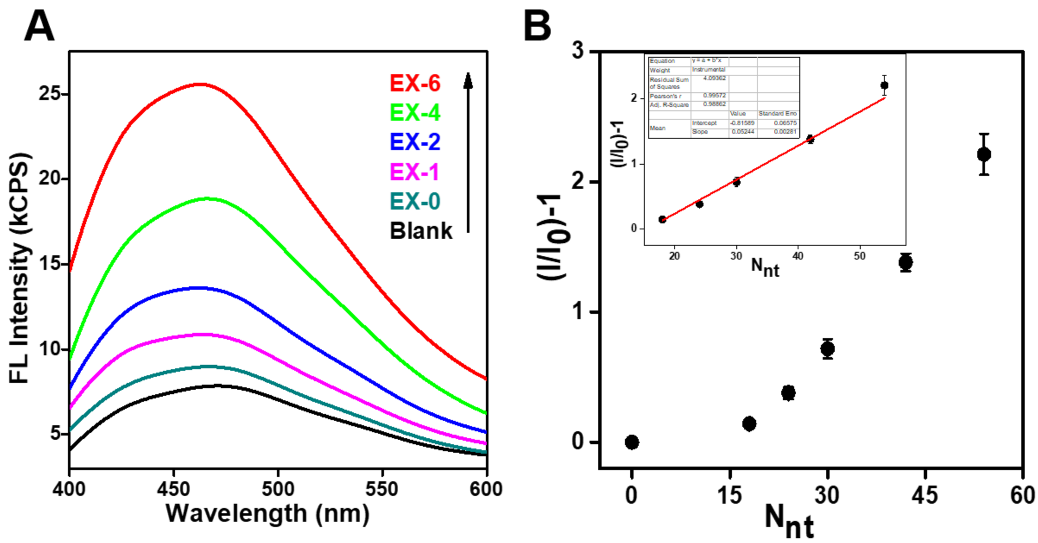

Supplementary Fig. 11. Specificity study about detection of telomerase activity from cancer cells extracts. The relative fluorescence intensity and fluorescence response of Silole-R in the absence (black) and presence of dNTPs, TP, Telomerase, telomerase extracted from 10000 AZT treated HeLa cells, telomerase extracted from 10000 HeLa cells, respectively. The concentration of Silole-R is 7.5 μM.

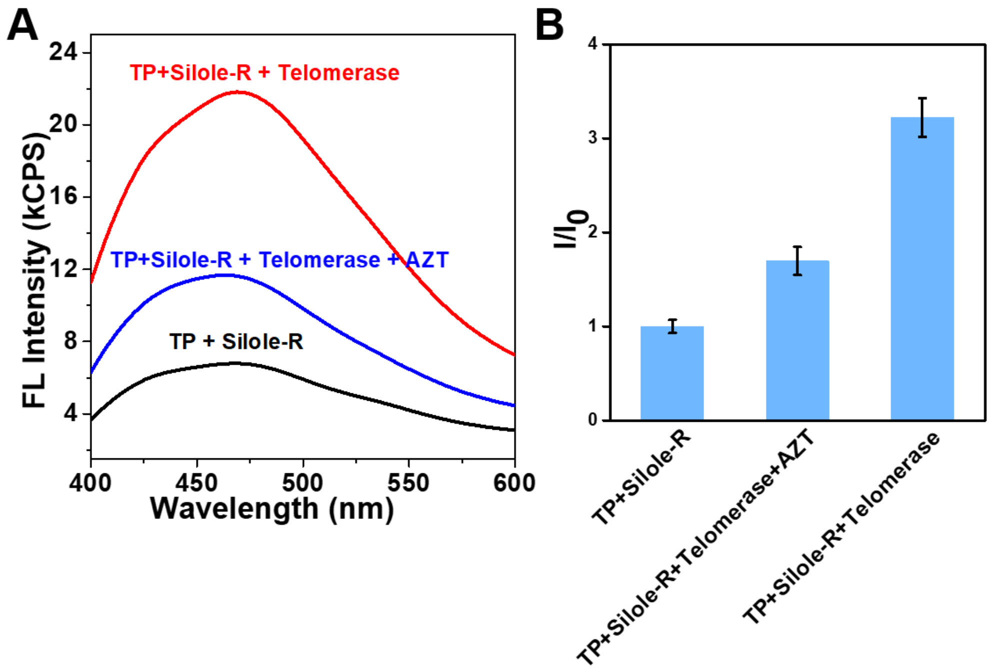

Supplementary Fig. 12. Fluorescence intensity of BSA (5 mg/L), trypsin (5 mM), T4 DNA Lignase (10 U), Bst DNA polymerase (15 U), thrombin(1 mM), lysis buffer, ATP (1 mM) and PBS were used in place of telomerase to carry out the extension reaction under the same experimental conditions.

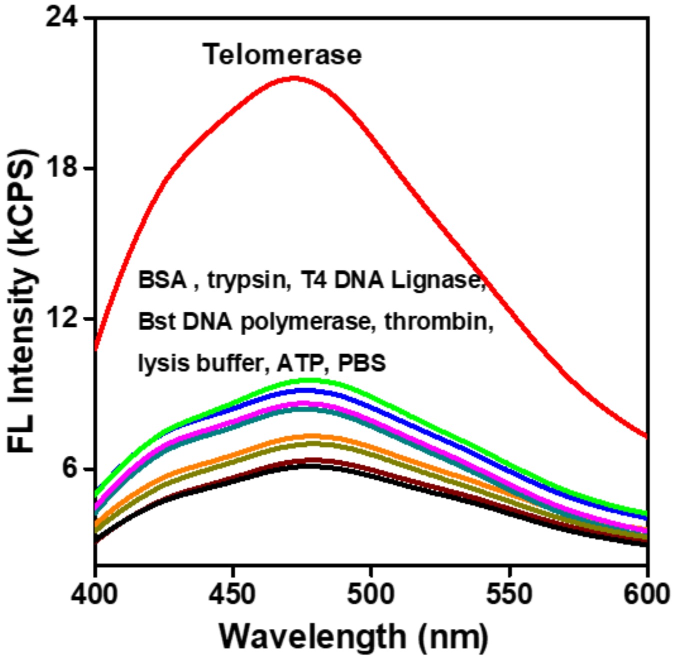

Supplementary Fig. 13. Non-denaturating PAGE analysis of telomerase extension assay. lane 1, DNA ladder; lane 2, 20 μM TP in the absence of cancer cell extract; lane 3-4, 20 μM TP in the presence of cell extract from 40000 and 50000 HeLa cells.

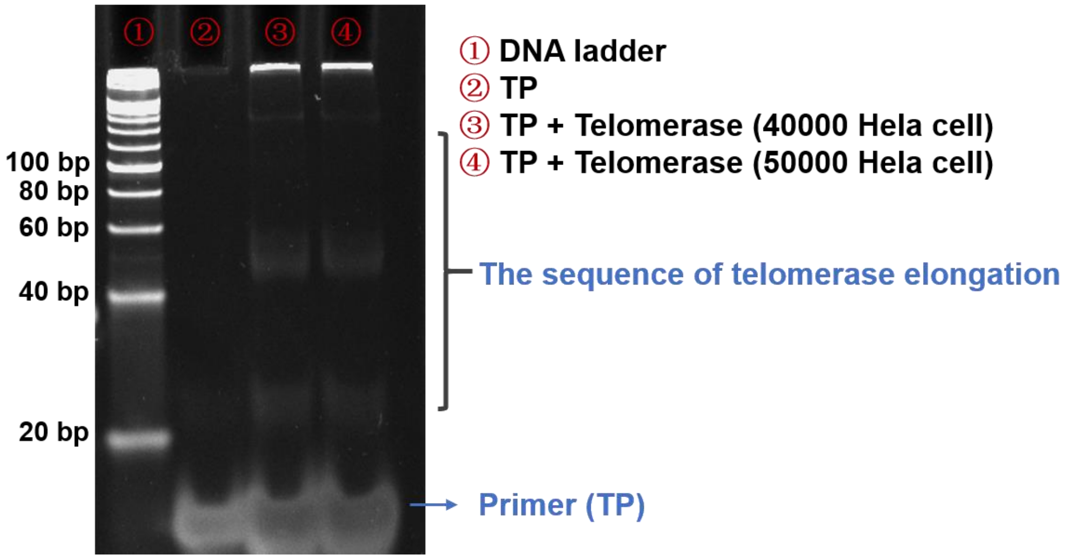

Supplementary Fig. 14. Gel electrophoresis of telomerase telomerase extend products (TRAP method) using TP or ACX are shown. The TRAP assay was performed on different amounts of cell telomerase extract. Land 1, DNA ladder; Land 2, TP + CX; Land 3, TP + CX + CHAPS lysis buffer, Land 4, TP + CX + 20000 HeLa cell telomerase extract; Land 5, TP + CX + 40000 HeLa cell telomerase extract.

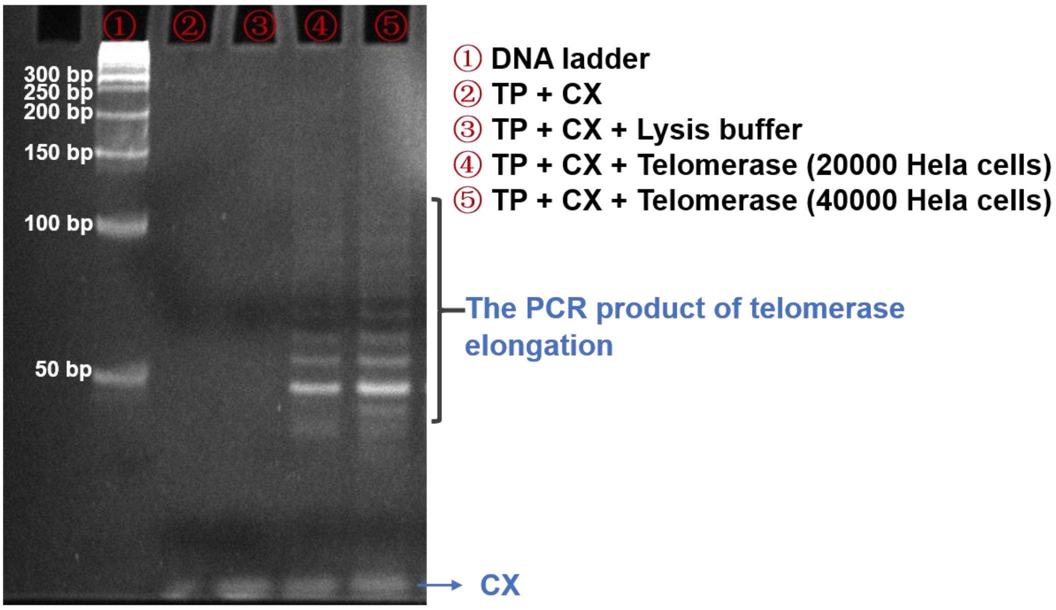

Supplementary Fig. 15. Percentage of cells in individual cycle phases after treatment with serum starvation, L-mimosine, thymidine and nocodazole respectively.

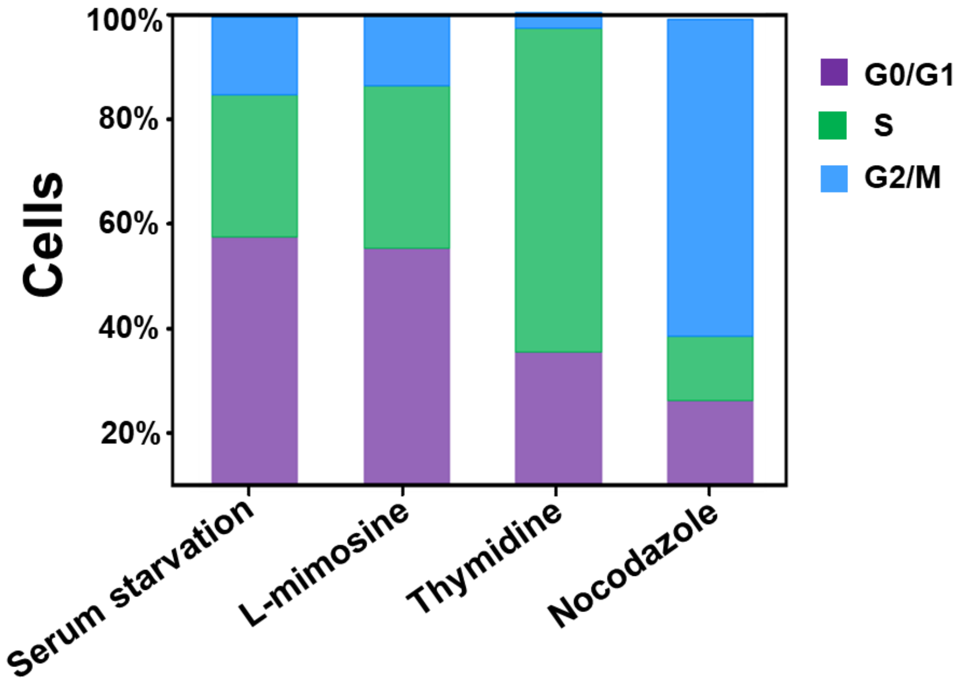

Supplementary Fig. 16. Fluorescence emission spectra of PyTPA-DNA incubation with TERT mRNA extracted from different phases of HeLa cells.

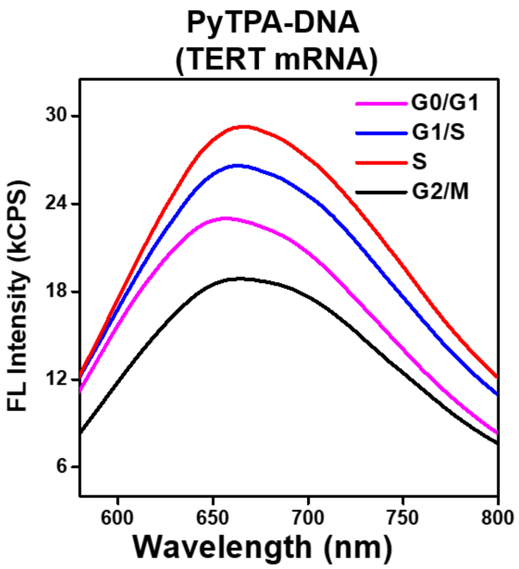

Supplementary Fig. 17. Fluorescence emission spectra of Silole-R in the presence of elongated TP and telomerase extracted from different phases of HeLa cells.

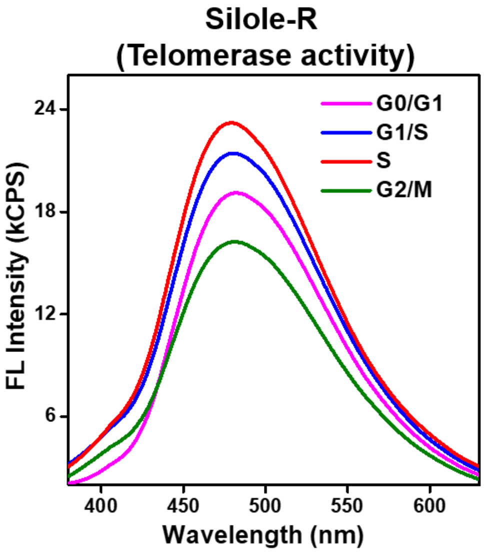

Supplementary Fig. 18. Telomerase extracted from different phases of HeLa cells and their corresponding telomerase activity were tested by quantitative TRAP assays.

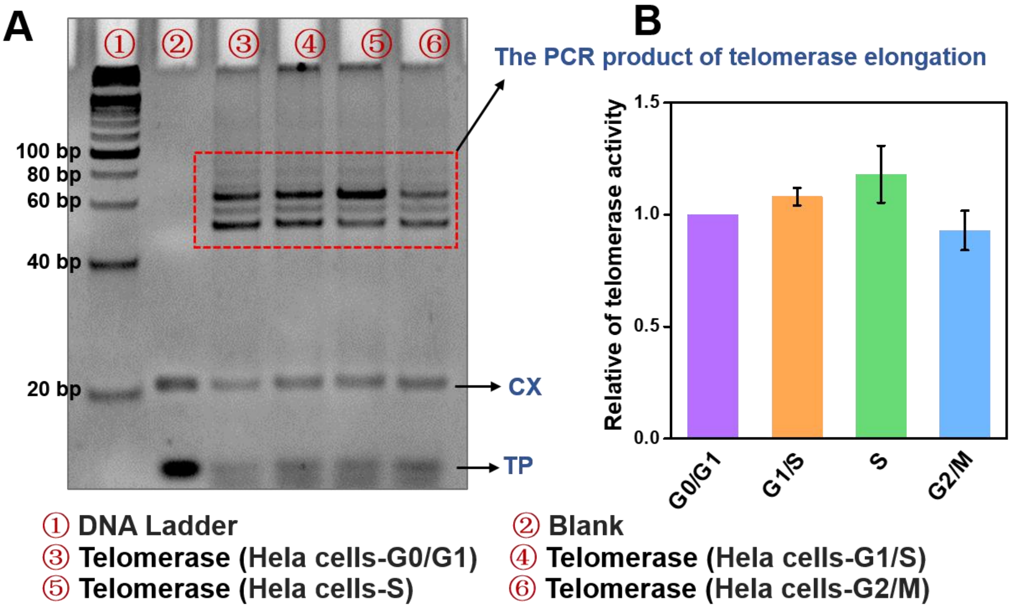

Supplementary Fig. 19. The cytotoxicity of PyTPA-DNA and Exo III were tested with HeLa cells by MTT assay. HeLa cells were incubated with PyTPA-DNA of different concentration (0, 2, 4, 6, 8, 10 μM) and Exo III of different concentration (0, 0.5, 1.0, 1.5, 2 U/μL) for 24 h.

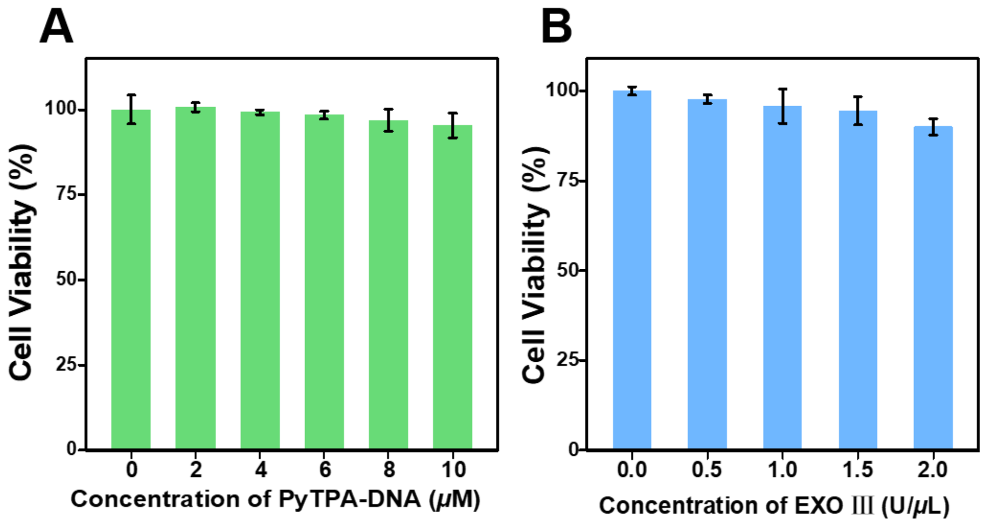

Supplementary Fig. 20. LSCM images of HeLa cells with PyTPA-DNA in the presence of Exo III at 37 °C for different times. 4.5 μmol/L PyTPA-DNA probes, 0.5 U/μL Exo III and 0.5 μmol/L were added to each cell-adhered dish and incubated for 0 ~ 2.5 h at 37 °C. The red fluorescence channel, excitation wavelength: 488 nm, emission collected: 580-700 nm. Scale bar: 20 μm.

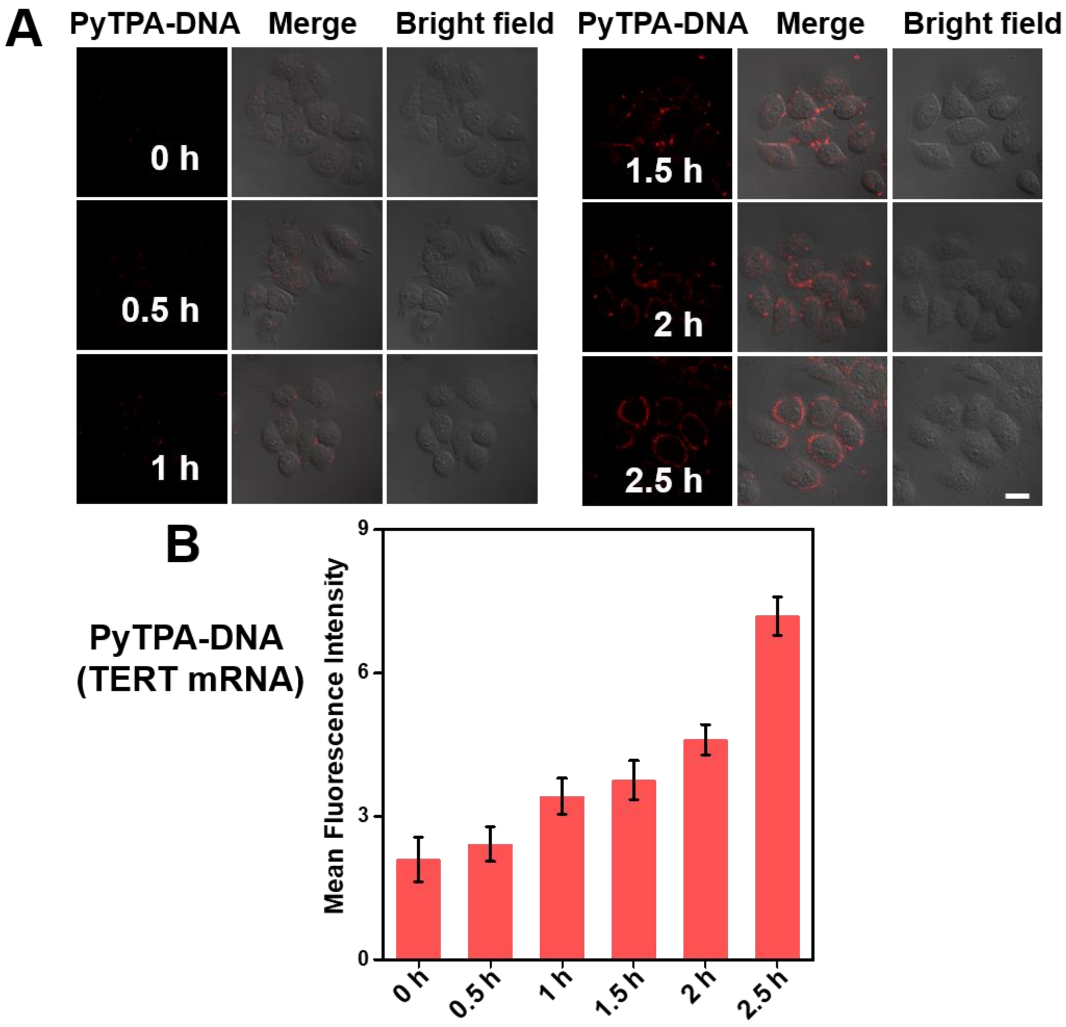

Supplementary Fig. 21. LSCM images of HeLa cells with EGCG stimulation. Different concentrations of EGCG (0, 60, 120 and 250 μg/mL) were added to each dish and incubated for 24 h. Afterwards, HeLa cells incubated with the PyTPA-DNA and exonuclease III for 2.5 h. 5 μmol/L PyTPA-DNA probes, 0.5 U/μL Exo III were added to each cell-adhered dish and incubated for 2.5 h at 37 °C. The red fluorescence channel, excitation wavelength: 488 nm, emission collected: 580-700 nm. Scale bar: 20 μm.

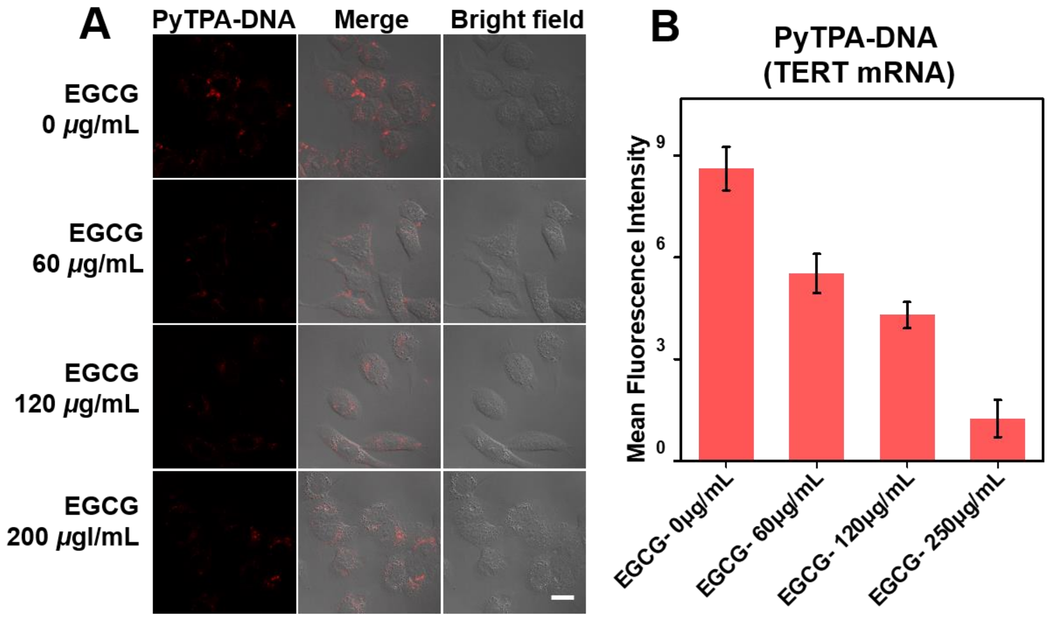

Supplementary Fig. 22. LSCM images of HeLa cells pretreated without or with AZT. (A) Confocal images of HeLa cells after transfection with 3.6 μM TP for 1 h and incubation with 5 μM Silole-R for 30 min. Cells were pretreated without or with 20 μM AZT for 48 h before transfection. (B) Fluorescence intensity in different cell groups of A. The blue fluorescence channel, excitation wavelength: 405 nm, emission collected: 400-500 nm. Scale bar: 20 μm.

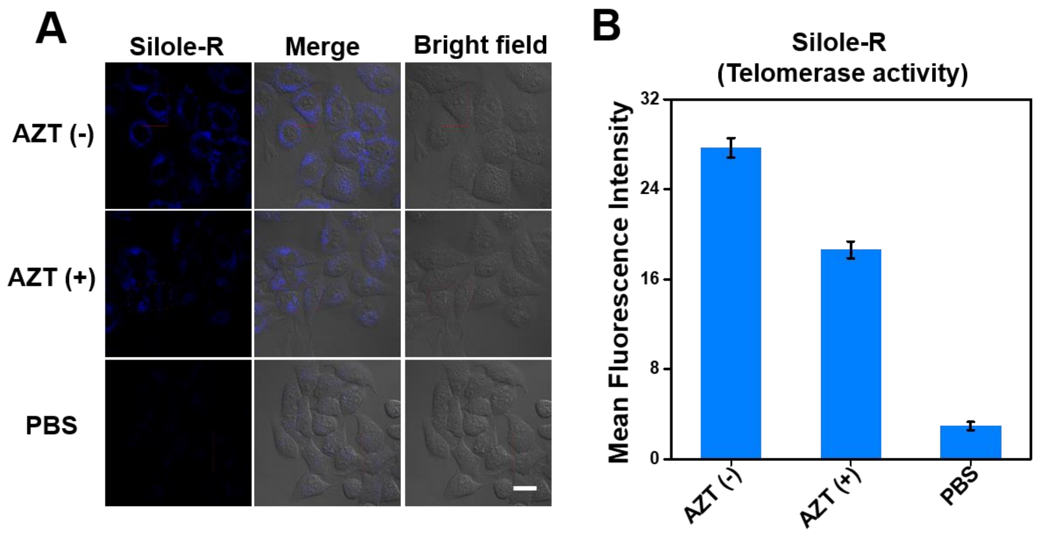

Supplementary Fig. 23. LSCM images of HeLa cells pretreated without or with different concentrations of EGCG. (A) Confocal images of HeLa cells after transfection with TP and then incubation with Silole-R. Cells were pretreated without or with different concentrations of EGCG for 48 h before transfection. (B) Fluorescence intensity in different groups of A. The blue fluorescence channel, excitation wavelength: 405 nm, emission collected: 400-500 nm. Scale bar: 20 μm.

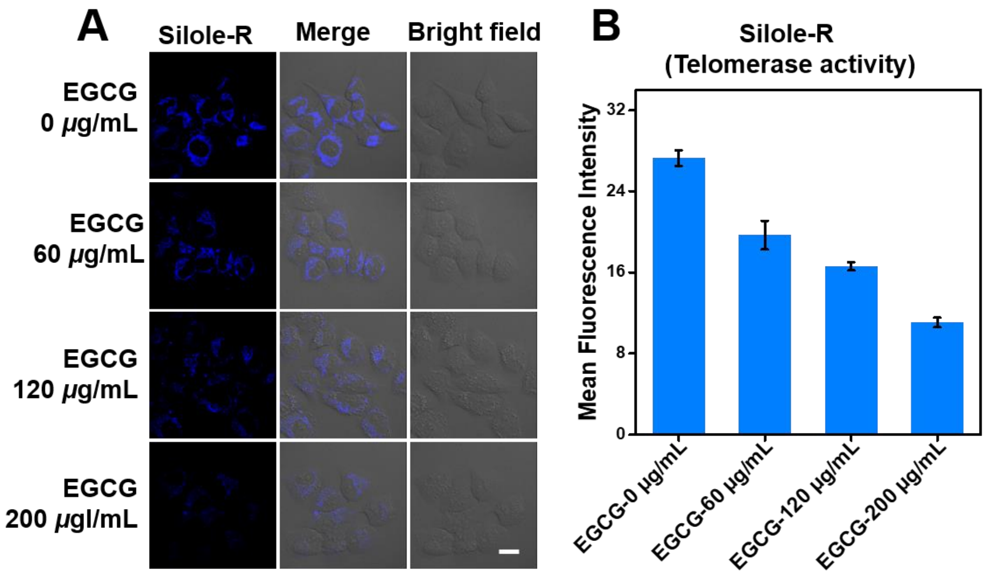

Supplementary Fig. 24. Time course of confocal images (fluorescence, merged and bright field) of HeLa cells arrested in different phases of cell cycle. (A) HeLa cells were incubated with the PyTPA-DNA probe solution and TP Silole-R solution for different times. (B-C) Corresponding fluorescence intensity of TERT and telomerase activity in different cells. This results proved this system were superb long-term and low-toxicity tracer for intracellular imaging of TERT mRNA and telomerase activity after 2.5 h incubation. The red fluorescence channel, excitation wavelength: 488 nm, emission collected: 580-700 nm. The blue fluorescence channel, excitation wavelength: 405 nm, emission collected: 400-500 nm. Scale bar: 20 μm.

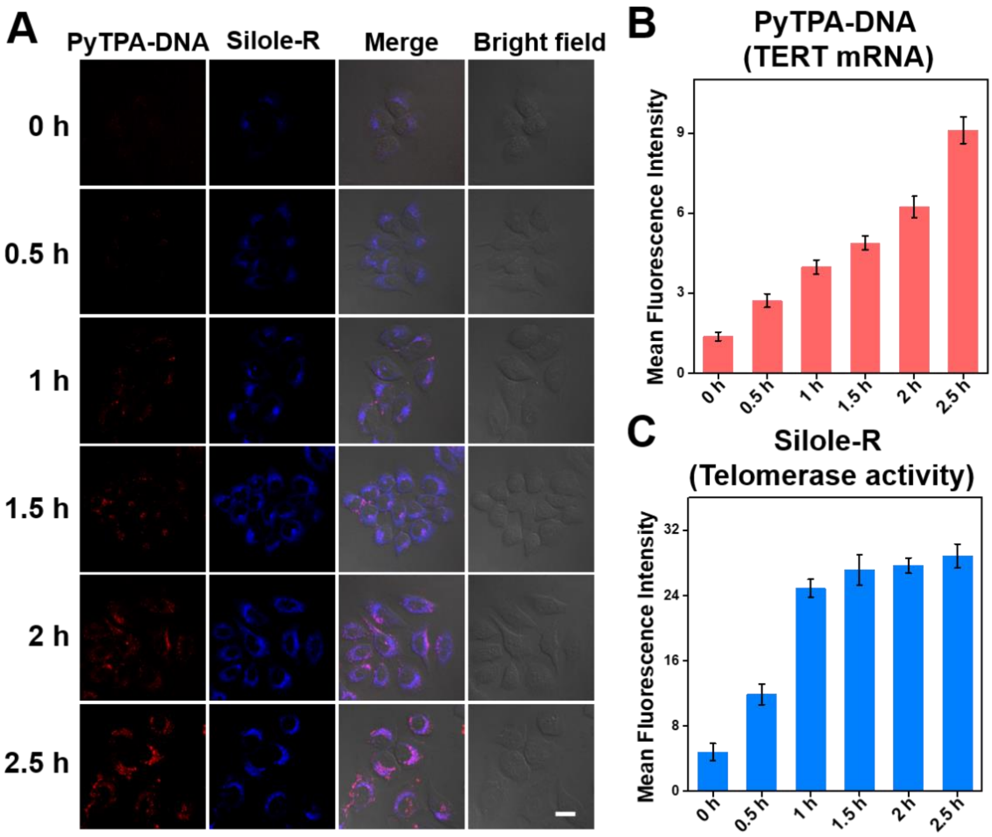

Supplementary Fig. 25. (A) LSCM images of HeLa cells synchronized in different cell cycles (control is the cell unsynchronized; G0/G1 phase; G1/S phase; S phase; G2/M phase) with PyTPA-DNA in the presence of Exo III at 37 °C. (B) Corresponding fluorescence intensity of TERT mRNA in different cell cycles. The red fluorescence channel, excitation wavelength: 488 nm, emission collected: 580-700 nm. Scale bar: 20 μm.

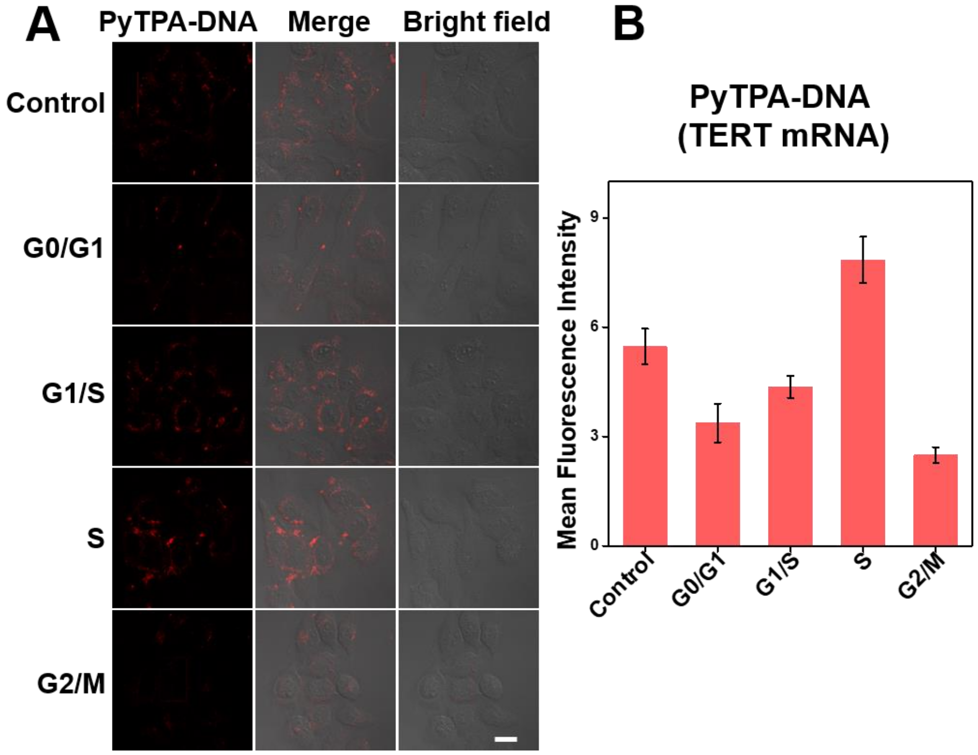

Supplementary Fig. 26. LSCM images of HeLa cells with TP for 1 h and then incubation with Silole-R at 37 ℃ in different cell cycle cells (control is the cell unsynchronized). The blue fluorescence channel, excitation wavelength: 405 nm, emission collected: 400-500 nm. Scale bar: 20 μm.

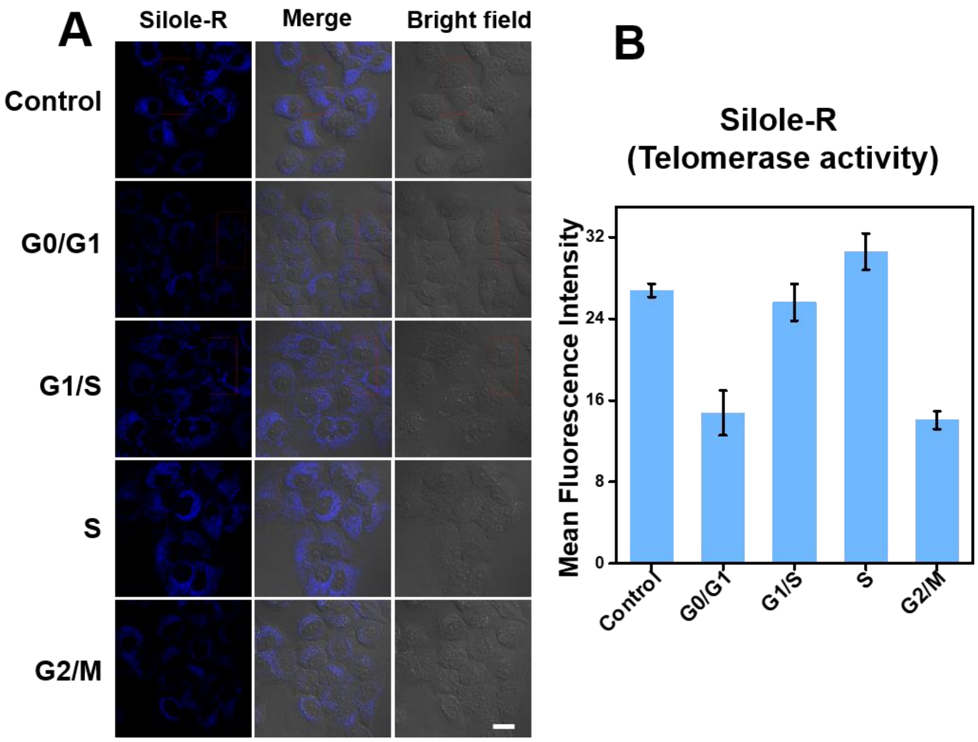

Supplementary Fig. 27. H&E staining displays extensive necrosis of tumor tissues in tumor groups, while there is little cell necrosis in benign tumour and the normal groups. Scale bar, 20 μm.

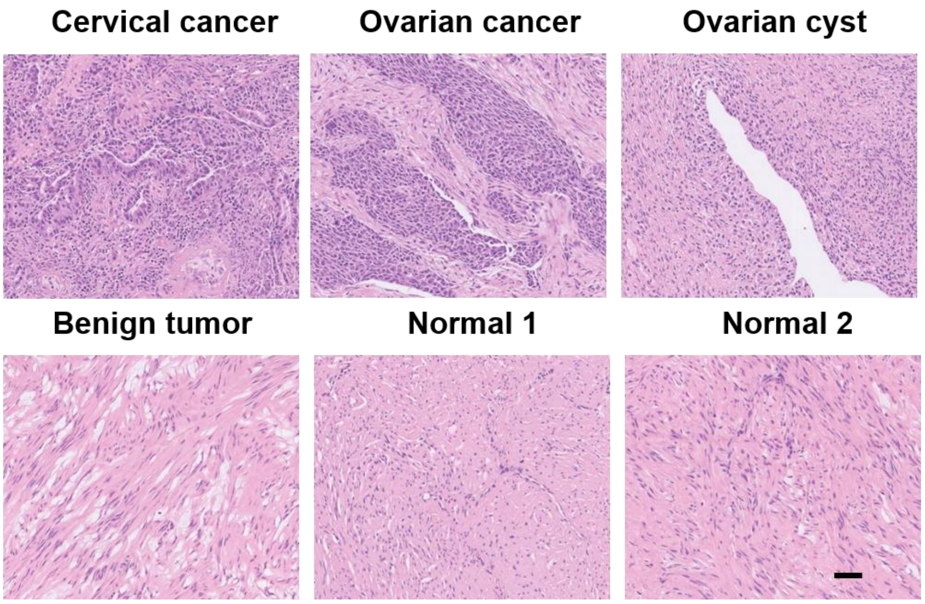

Supplementary Fig. 28. Gene expression profiles of well-characterized cell cycle genes. The expression profiles are shown for 19 well-characterized genes whose expression peaks in each phase of the cell cycle. In each time course, RNA of HeLa cells were collected for points after the synchronous arrest. The four genes representing each cell cycle phase were averaged to create five different "ideal expression profiles" (G0/G1, G1/S, S and G2/M). CCNE1 and E2F1-2 expression at the G1/S boundary was accompanied. RRM2 and CCNE2 (DNA metabolism genes) were represented at the beginning of S phase. Maximum expression of the CCNA2 and CCNB1 (mitotic cyclins) were observed at the expected times of G2/M phase.

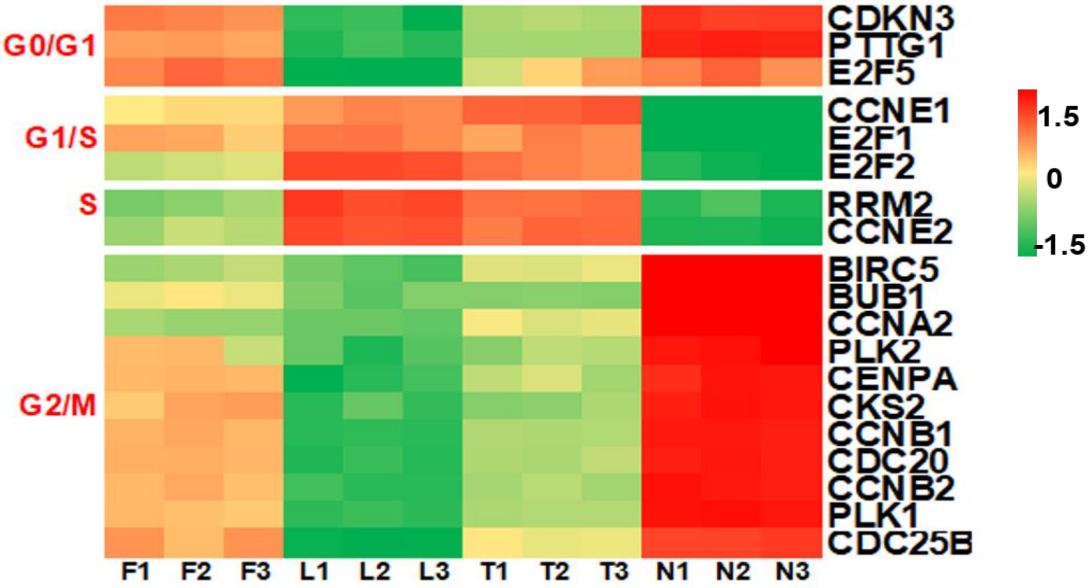

Supplementary Fig. 29. The expression of TERT in different phases of cell cycle.

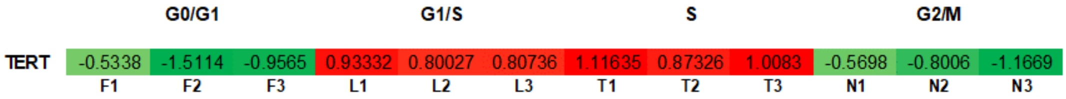

	G0/G1			G1/S			S			G2/M		
	F1	F2	F3	L1	L2	L3	T1	T2	T3	N1	N2	N3
TERT	-0.5338	-1.5114	-0.9565	0.93332	0.80027	0.80736	1.11635	0.87326	1.0083	-0.5698	-0.8006	-1.1669

Supplementary Fig. 30. Profiling of different expression genes (DEGs) in G0/G1 and G1/S cell cycles. (A) Volcano plots for DEGs from comparison in different groups. (B) Clustered heatmap for DEGs from different groups with rows representing DEGs and columns representing samples. Heatmap showing the expression pattern of the core 20 genes during cell cycle. Red Dots represent the change (red, up-regulation; green, downregulation; black, no change).

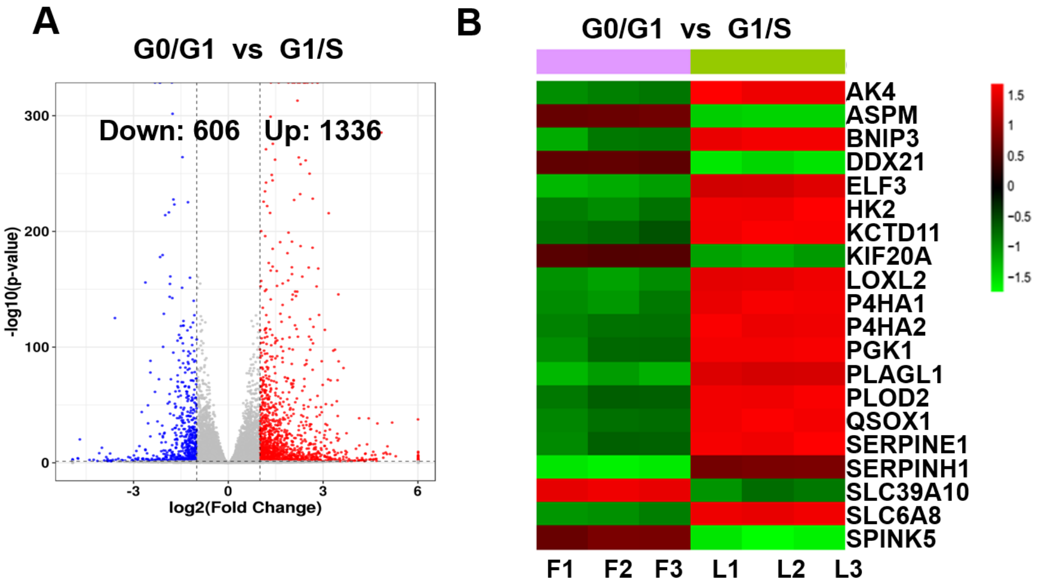

Supplementary Fig. 31. Profiling of different expression genes (DEGs) in G0/G1 and S cell cycles. (A) Volcano plots for DEGs from comparison in different groups. (B) Clustered heatmap for DEGs from different groups with rows representing DEGs and columns representing samples. Heatmap showing the expression pattern of the core 20 genes during cell cycle. Red Dots represent the change (red, up-regulation; green, downregulation; black, no change).

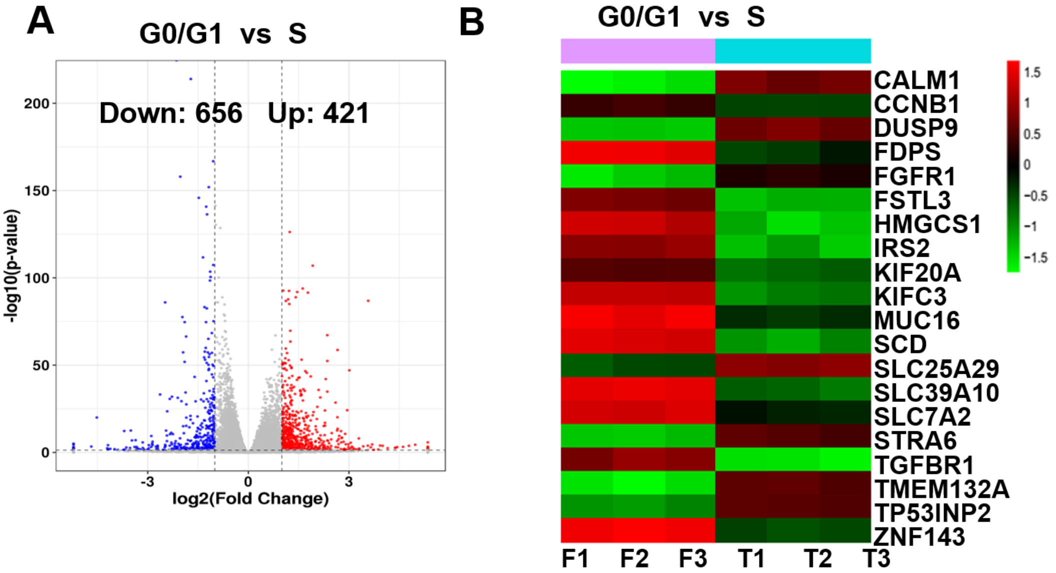

Supplementary Fig. 32. Profiling of different expression genes (DEGs) in G0/G1 and G2/M cell cycles. (A) Volcano plots for DEGs from comparison in different groups. (B) Clustered heatmap for DEGs from different groups with rows representing DEGs and columns representing samples. Heatmap showing the expression pattern of the core 20 genes during cell cycle. Red Dots represent the change (red, up-regulation; green, downregulation; black, no change).

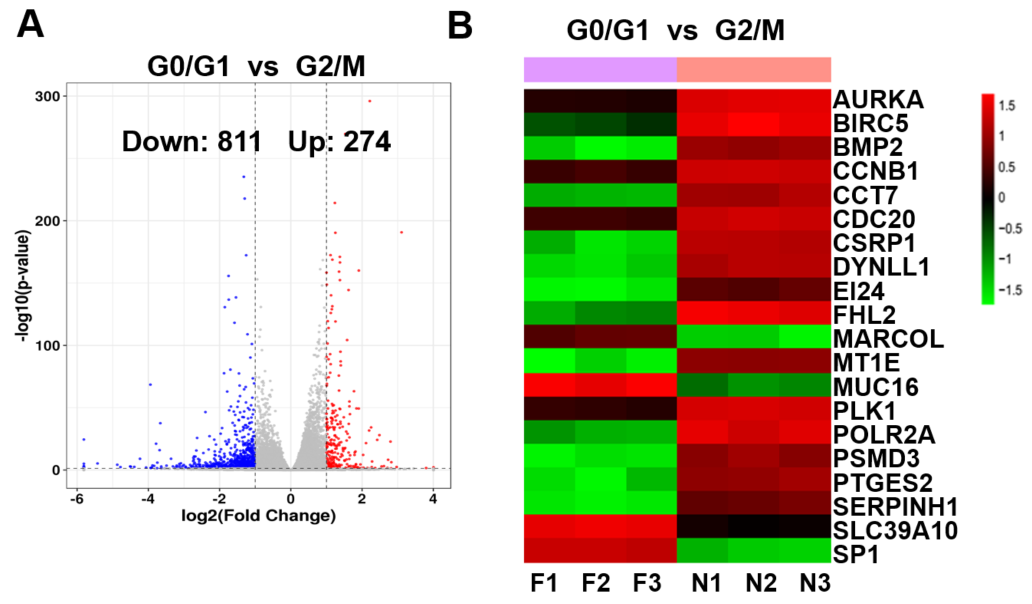

Supplementary Fig. 33. Significantly altered DEGs in the G1/S phase compared with G0/G1 phase were functionally annotated. Several major pathways were related to system development, multicellular organism development and cellular developmental process.

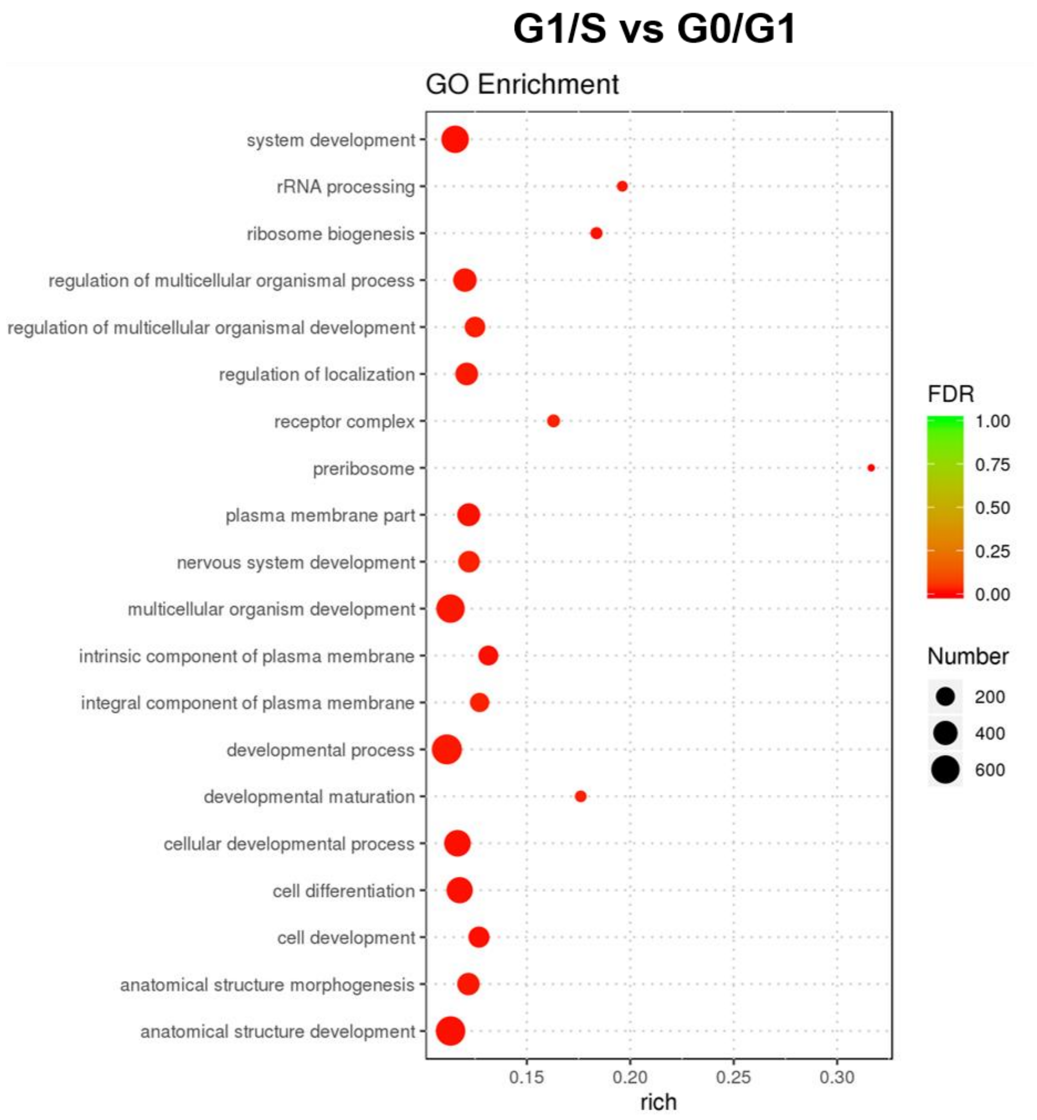

Supplementary Fig. 34. Gene Ontology (GO) analysis of the biological process (BP) terms of genes upregulated and downregulated in S vs G0/G1 of HeLa cell lines. In S phase, the altered DEGs compared with G0/G1 group using GO analysis were mainly related to the plasma membrane and cell periphery and cell differentiation, likely the most relevant to the mechanism of cell synthesis and replication.

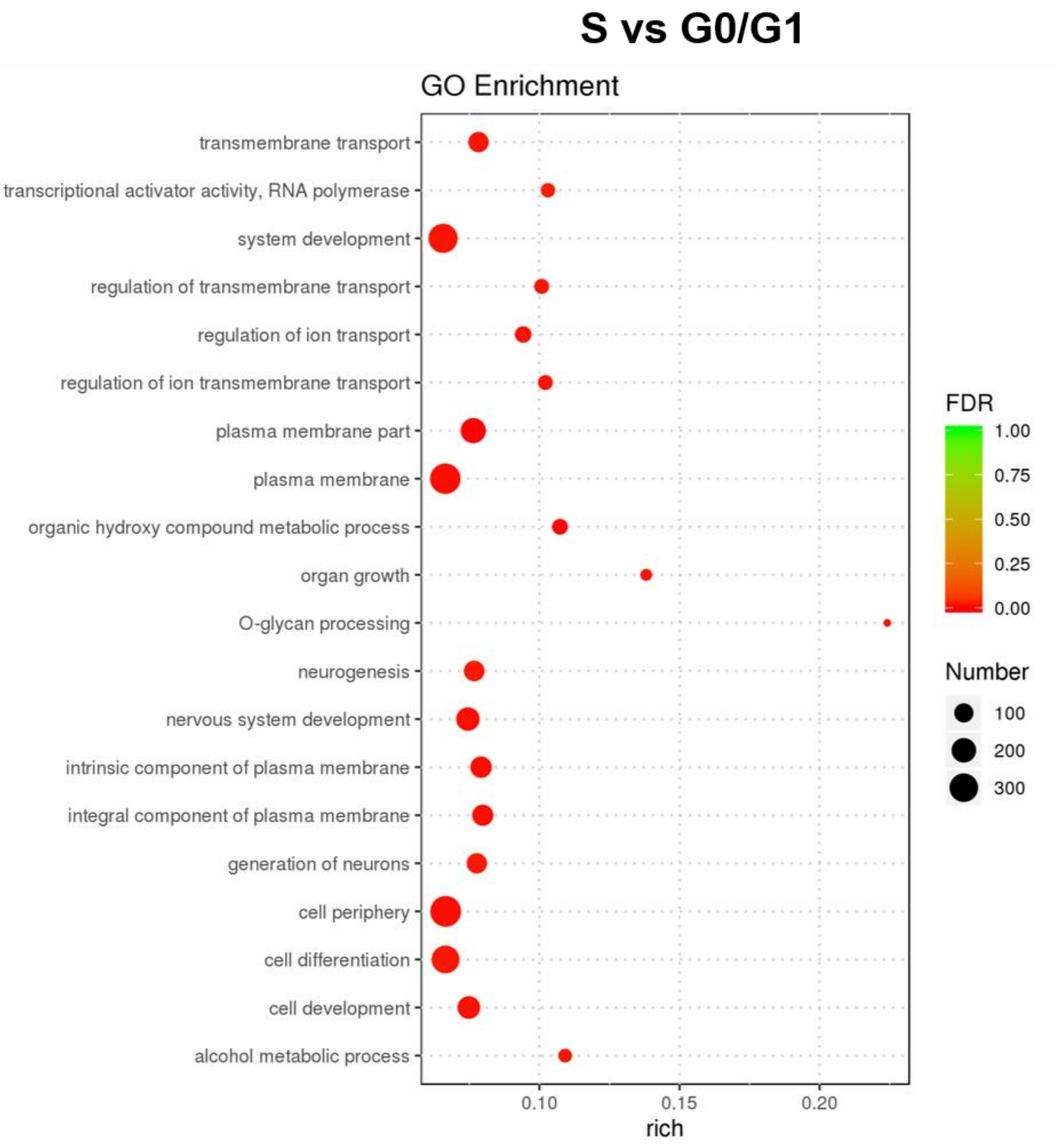

Supplementary Fig. 35. Gene Ontology (GO) analysis of the biological process (BP) terms of genes upregulated and downregulated in G2/M vs G0/G1 of HeLa cell lines. Compared with the G0/G1 phase, G2/M phase with several major pathways related to plasma membrane part, plasma membrane bounded cell projection organization and cell projection organization.

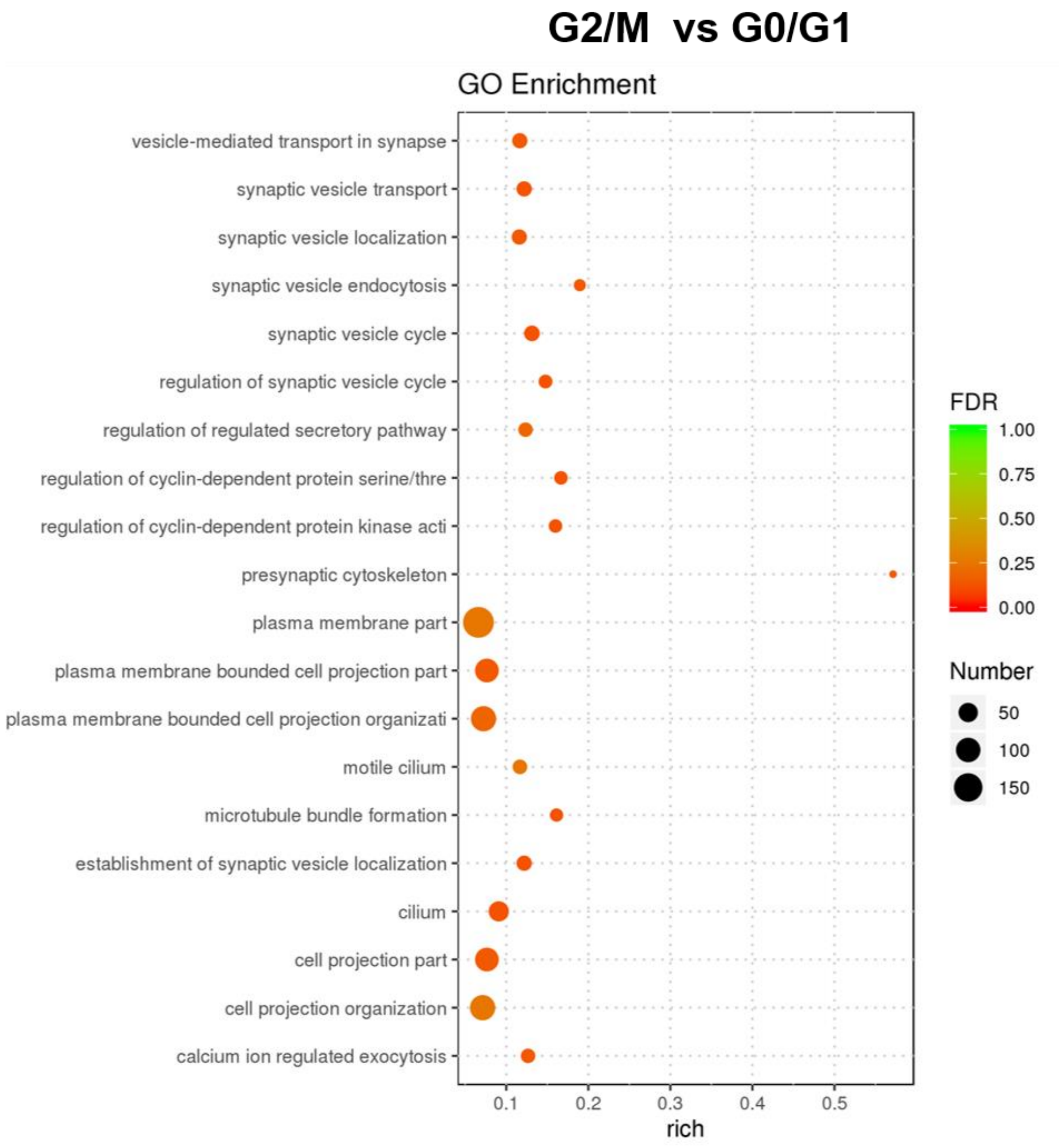

References

1. Jackman J and O'Connor PM. Methods for synchronizing cells at specific stages of the cell cycle. *Curr Protoc Cell Biol* 2001; **Chapter 8**: Unit 8.3.
2. Kim C, Lim Y and Chul B *et al*. Biochimica et biophysica acta regulation of post-translational protein arginine methylation during HeLa cell cycle. *Biochim Biophys Acta* 2010; **1800**: 977-85.
3. Martin M. Cutadapt removes adapter sequences from high-throughput sequencing reads. *Embnet Journal*, 2011; **17**:1-3 .
4. Langmead B and Salzberg S L. Fast gapped-read alignment with Bowtie 2. *Nature Methods*, 2012, **9**:357-359.
5. Jouni Sirén, Niko Välimäki and Veli Mäkinen. Indexing graphs for path queries with applications in genome research. *IEEE/ACM Trans Comput Biolo Bioinformatics*, 2014; **11**:375-88.
6. Simon Anders, Paul Theodor Pyl and Wolfgang Huber. HTSeq-a Python framework to work with high-throughput sequencing data. *Bioinformatics*, 2015; **31**:166-9.
7. Anders S and Huber W. Differential expression analysis for sequence count data. *Genome Biol*, 2010; **11**:1-12.
